# Myocardial ischemia‐reperfusion induced cardiac extracellular vesicles harbour proinflammatory features and aggravate heart injury

**DOI:** 10.1002/jev2.12072

**Published:** 2021-02-23

**Authors:** Xinyu Ge, Qingshu Meng, Lu Wei, Jing Liu, Mimi Li, Xiaoting Liang, Fang Lin, Yuhui Zhang, Yinzhen Li, Zhongmin Liu, Huimin Fan, Xiaohui Zhou

**Affiliations:** ^1^ Research Center for Translational Medicine Shanghai East Hospital Tongji University School of Medicine Shanghai P.R. China; ^2^ Shanghai Heart Failure Research Center Shanghai East Hospital Tongji University School of Medicine Shanghai P.R. China; ^3^ Institute of Integrated Traditional Chinese and Western Medicine for Cardiovascular Chronic Diseases Tongji University School of Medicine Shanghai P.R. China; ^4^ Department of Cardiothoracic Surgery Shanghai East Hospital Tongji University School of Medicine Shanghai P.R. China; ^5^ Department of Heart Failure Shanghai East Hospital Tongji University School of Medicine Shanghai P.R. China; ^6^ Department of Respiratory Medicine Shanghai East Hospital School of Medicine Tongji University Shanghai P.R. China; ^7^ Department of Ultrasound Shanghai East Hospital School of Medicine Tongji University Shanghai P.R. China

**Keywords:** extracellular vesicles, heart injury, inflammation, ischemia‐reperfusion injury, macrophage polarization, miRNAs

## Abstract

Extracellular vesicles (EVs) curb important biological functions. We previously disclosed that ischemia‐reperfusion (IR) induces increased release of EVs (IR‐EVs) in the heart. However, the role of IR‐EVs in IR pathological process remains poorly understood. Here we found that adoptive transfer of IR‐EVs aggravated IR induced heart injury, and EV inhibition by GW4869 reduced the IR injury. Our in vivo and in vitro investigations substantiated that IR‐EVs facilitated M1‐like polarization of macrophages with increased expression of proinflammatory cytokines. Further, we disclosed the miRNA profile in cardiac EVs and confirmed the enrichment of miRNAs, such as miR‐155‐5p in IR‐EVs compared to EVs from the sham heart (S‐EVs). In particular, IR‐EVs transferred miR‐155‐5p to macrophages and enhanced the inflammatory response through activating JAK2/STAT1 pathway. Interestingly, IR‐EVs not only boosted the local inflammation in the heart, but even triggered systemic inflammation in distant organs. Taken together, we newly identify an IR‐EVs–miR‐155‐5p**–**M1 polarization axis in the heart post IR. The EVs derived from IR‐injured heart contribute to both local and systemic inflammation. Importantly, EV inhibition by GW4869 is supposed to be a promising therapeutic strategy for IR injury.

## 1 INTRODUCTION

Acute myocardial infarction (MI) is a global issue with high incidence and mortality (Yeh et al., 2010). Rapid recovery of the blocked coronary flow represents the most effective strategy to reduce the size of myocardial infarction and improve the cardiac function. However, reperfusion lead to irreversible myocardial injury (Yellon & Hausenloy, 2007), which is known as myocardial ischemia‐reperfusion (IR) injury (Frank et al., 2012). Myocardial IR exacerbates the cardiac dysfunction and increases the incidence of adverse prognosis. However, the mechanism of IR injury remains elusive, and effective treatments are still lacking.

**STATEMENT OF SIGNIFICANCE**

Recent studies highlight the vital role of extracellular vesicles (EVs) as cell‐to‐cell communication vehicles in the development of various diseases. However, little is known about the feature and function of tissue‐derived EVs. The present study disclosed a cardiac EV‐based mechanism involved in myocardial ischemia‐reperfusion (IR) injury. IR induced EVs (IR‐EVs) can promote inflammatory response and aggravate the heart inj ury through IR‐EVsmiR‐155‐5pM1 polarization axis. IR‐EVs contribute to both the local and systemic inflammation, which supports a role for IR‐EVs in the initiation of inflammatory phase in the heart as well as a timely communication between the heart and the other organs. The protective effect of GW4869 in the cardiac IR inj ury suggests that early inhibition of the detrimental EVs should be a promising strategy.

Inflammation is a fundamental biological process for maintaining homeostasis in the body. However, excessive inflammatory response contributes to tissue injury. After MI, early inflammation is closely related to the final size of the infarct area (Jose et al., 2014). Macrophages, as the vital effectors and regulators of inflammatory cascade triggered by MI, are involved in pathogens clearance and promotion of adaptive immune responses. Polarization of macrophages, such as classical activation (M1) and alternative activation (M2), is the key to achieve these functions (Murray, 2016). M1 macrophages, known as the proinflammatory type, are important in phagocytosis and secretion of pro‐inflammatory cytokines, whereas M2 macrophages are mainly involved in the regulation of inflammation and the repair of injured tissues. Modulation of pro‐inflammatory monocytes or M1 macrophages after MI has cardioprotective effect (Courties et al., 2014; Harel‐Adar et al., 2011). In contrast, promoting the polarization of M2 macrophages can alleviate inflammation and prevent poor ventricular remodelling after MI (Fan et al., 2018; Weirather et al., 2014). Therefore, polarization status of macrophages during MI and IR injury is regarded as a potential therapeutic target.

Extracellular vesicles (EVs) are cell‐derived nanosized vesicles with double‐layer lipid membrane (Abels & Breakefield, 2016). Increasing evidence confirmed the vital role of EVs as cell‐to‐cell communication vehicles (Tkach & Théry, 2016). Recent studies have disclosed their function in inflammation regulation and confirmed their participation in the development of various diseases by regulating the polarization of macrophages (Lv et al., 2020; Wang et al., 2020; Yin et al., 2020). We previously demonstrated the significantly increased release of cardiac EVs post IR (Ge et al., 2019). However, the role of IR induced EVs (IR‐EVs) remains to be disclosed. A large number of studies have indicated that non‐coding RNA (ncRNA) plays an important role in cardiovascular disease (Beermann et al., 2016), especially miRNAs (Karakas et al., 2016; Leistner et al., 2016; Mcdonald et al., 2015; Zhang et al., 2017). EVs can transfer specific molecules such as miRNAs (Bang et al., 2014; Okoye et al., 2014) into target cells to regulate their function, and thus participate in the pathogenesis. In murine MI models, myocardial microRNAs in circulating EVs mobilized bone marrow progenitor cells, mediating a systemic response to cardiac injury (Cheng et al., 2019). However, whether and how cardiac IR‐EVs and the EV‐delivered miRNAs exert their roles during IR injury are poorly understood. The present study newly demonstrated that IR‐EVs aggravated the heart injury post IR, and early inhibition of IR‐EVs could be a promising strategy. Interestingly, IR‐EVs not only contributed to the sterile inflammation in the IR‐injured heart, but even induced systemic inflammation in distant organs. Especially, we identified the miRNA profile in cardiac EVs, and disclosed the IR‐EVs–miR‐155‐5p–M1 polarization axis during cardiac IR.

## 2 METHODS

### 2.1 Animals

Wild‐type C57BL/6J mice (male, 8–10 weeks old) were purchased from SLAC Laboratory Animal co., Ltd. (Shanghai, China). Mice were kept in the specific‐pathogen‐free (SPF) room with constant temperature (23‐24°C), humidity (55 ± 5%) and light (12 h light‐dark cycle). All the mice had free access to food and water. The in vivo manipulations were approved by the Institutional Animal Care and Use Committee of Tongji University (Number: TJLAC‐019‐148). Computer‐generated random numbers were used for random grouping in the present study.

### 2.2 Myocardial IR model

Murine myocardial IR model was conducted according to our previous study (Ge et al., 2019). Mice in GW4869 group received intraperitoneal injection of GW4869 (2.5 mg/kg, Sigma‐Aldrich) 1 h before IR surgery. After anesthetizing with 1.5% pentobarbital (50 mg/kg body weight, Sigma‐Aldrich), chest cavity was opened at the 4th intercostal space under mechanical ventilation. The left anterior descending (LAD) coronary artery was ligated for 45 min with an 8‐0 silk suture. Once removing the silk suture, intracardiac injection of 50μL IR‐EVs (2×10^8^/μL) or PBS was performed through the left ventricle. After closing thoracic incision, the mice were placed on a heated blanket until recovery from anaesthesia.

### 2.3 EVs isolation

Cardiac EVs isolation was performed using differential centrifugation as previously described (Ge et al., 2019). Briefly, the heart was perfused with Phosphate‐buffered saline (PBS). Left ventricle tissues subjected to IR injury were removed and digested in 0.1% type II collagenase (Sigma‐Aldrich, USA) at 37°C for 30 min. The digested tissue was centrifuged at 300 × *g* for 5 min to remove the tissues and cells. The supernatant was centrifuged at 2000 × *g* for 10 min, and then 10000 × *g* for 10 min at 4°C. The supernatant was centrifuged at 120,000 g for 2 h at 4°C to pellet all EVs (Optima L‐100XP Ultracentrifuge, Beckman Coulter). After one wash with PBS, the EVs were obtained and resuspended in 100 μL PBS.

### 2.4 Electron microscopy

The fresh‐isolated cardiac EVs were fixed with 2.5% glutaraldehyde stationary liquid. EV suspensions were loaded onto 200 Mesh carbon‐coated formvar grids for 5 min, and then stained with 2% phosphotungstic acid for 5 min at room temperature. EV samples were detected using a transmission electron microscope (TEM; Hitachi, HT7700).

### 2.5 Nanoparticle tracking analysis (NTA)

The particle size and concentration of the EVs were tested using the ZetaView NTA technique by Particle Metrix (Meerbusch, Germany) with three times replicates.

### 2.6 Echocardiography

Cardiac function was evaluated by echocardiography (VisualSonics, Canada). The mice were anesthetized with isoflurane after depilation. The left ventricular end diastolic diameter (LVEDD) and left ventricular end systolic diameter (LVESD) were measured in two‐dimensional long axis views. The left ventricular ejection fraction (EF), fraction shortening (FS), left ventricular mass, and left ventricular contraction volume were calculated for cardiac function assessment.

### 2.7 Myocardial enzymes detection and circulating leukocytes collection

One day after IR surgery, blood was collected in heparin‐containing collection tubes. The plasma was isolated by centrifugation (2000 g, 4˚C, 10 min) to detect aspartate transaminase (AST), lactate dehydrogenase (LDH), creatine kinase (CK) and creatine kinase isoenzyme (CK‐MB) levels using Beckmann AU680 (Beckman Coulter, Inc.) according to manufacturer's instructions. The layered circulating leukocytes under plasma were transferred to a new tube. After erythrocyte lysis, circulating leukocytes were collected for further investigation.

### 2.8 2, 3, 5‐triphenyltetrazolium chloride (TTC) staining

Mice were sacrificed after a 24‐h reperfusion period. After PBS washing, the heart was frozen in a ‐20°C refrigerator for 20 min. The heart was immediately cut into 1‐mm‐thick slices (typically, five slices per heart) and stained with 2% (wt/vol) TTC (Sigma‐Aldrich) at 37°C for 15 min. Images were analyzed by Image‐Pro Plus (Media Cybernetics).

### 2.9 Immunofluorescence staining

Mouse heart was fixed in 4% paraformaldehyde. The fixed heart tissues were dehydrated and embedded in paraffin. For immunofluorescence staining, heart sections were incubated with anti‐CD45 and anti‐F4/80 antibody (1:100, Cell Signaling Technology, USA) at 4°C overnight. After 3 times wash with PBS, fluorescein‐isothiocyanate‐conjugated secondary antibodies (1:1,000, Cell Signaling Technology, USA) were incubated at room temperature in the dark for 1 h. After DAPI (Sigma‐Aldrich, USA) staining, fluorescence images were captured under the fluorescence microscope (Leica, Wetzlar, Germany).

### 2.10 Confocal imaging

EVs were incubated with the lipophilic dye 1,1′‐dioctadecyl‐3,3,3′,3′‐tetramethylindocarbocyanine perchlorate (DiI; 10μM, Beyotime Biotechnology) for 5 min in dark at room temperature according to the manufacturer's directions. DiI‐labelled EVs were co‐cultured with macrophages for 24 h. Cells were washed with PBS and then fixed with 4% (w/v) paraformaldehyde for 15 min. DAPI (Sigma‐Aldrich, USA) were used for nuclei staining. Confocal images were obtained using Leica SP8 System (Leica, Wetzlar, Germany).

### 2.11 Bioluminescence imaging

IR‐EVs were prestained with the liposomal dye DiR (Thermo Scientific, USA) according to manufacturer's instructions. Intracardiac or tail vein injection of DiR‐labelled EVs was performed as indicated in the study. The organs including heart, lung, liver, kidney, spleen and brain were excised and rinsed in PBS 24 h later. Bioluminescence imaging was performed using IVIS imaging system (Xenogen, USA).

### 2.12 Isolation and cultivation of murine bone marrow derived macrophages (BMDMs) and peritoneal macrophages (PMφ)

Bone marrow cells were obtained by flushing the femurs of C57BL/6J mice with Dulbecco's modified Eagle's medium (DMEM) medium (Gibco, Eggenstein, Germany). Cells were collected, centrifuged at 300 × *g* for 5 min, then suspended in DMEM (10% heat inactivated FBS (Gibco, Eggenstein, Germany); 20% L929 supernatant) and cultured at 37˚C and 5% CO_2_ for 7 days.

PMφ cells were isolated by peritoneal lavage on the third day after intraperitoneal injection of 3% thioglycollate medium (Merck KGaA, Darmstadt, Germany). Cells were cultured in Roswell Park Memorial Institute (RPMI) 1640 medium (Gibco, Eggenstein, Germany) with 10% heat inactivated FBS and 1% Penicillin‐Streptomycin (Gibco, Eggenstein, Germany).

### 2.13 Phagocytosis assay

Phagocytic function of macrophages was detected using the phagocytosis assay kit (Invitrogen, CA, USA) according to the manufacturer's instructions.

### 2.14 Flow cytometry of cardiac macrophages

One day after IR, hearts were perfused with pre‐cold PBS. Left ventricle tissues subjected to IR injury were removed and dissociated with gentleMACS dissociator (Miltenyi Biotec, USA). The digestion was performed in 5 ml HBSS buffer contained Collagenase II (Worthington, 1.5 mg/ml), Collagenase IV (Worthington, 1.5 mg/ml) and DNase I (Sigma, 60U/ml) at 37℃ for 30 min at a speed of 200 rpm. The resulting suspension was filtered (70 μm) to generate a single‐cell suspension. The suspension was centrifuged at 300 × *g* for 5 min. Cardiac cells were then resuspended in DMEM media (supplemented with 10% FBS and 1% Penicillin‐Streptomycin) and cultured for 2 h (37°C, 5% CO2). Cells were then washed with PBS and cardiac macrophages were enriched in the adherent cells (de Couto et al., 2017). Cells were collected and incubated with flow cytometry antibodies, including FITC‐CD4, PE‐F4/80, PerCP‐Cy5.5‐CD86 and PE‐Cy7‐CD206 (all from BioLegend, USA) at 4°C in the dark for 15 min. After PBS washing, the phenotype of cardiac macrophages was detected by BD FACSCanto II flow cytometer (BD Bioscience, USA).

### 2.15 In vitro microRNA transfection

All the miRNA mimics and inhibitors used in the study were synthesized by Sangon Biotech, Shanghai, China. MicroRNA transfection was performed using riboFECT CP transfection regent (RiboBio Co., LTD, Guangzhou, China) following the manufacturer's directions.

### 2.16 In vivo microRNA administration

The agomiR‐155 and miRNA scramble (agomiR‐Sc) were synthesized by Sangon Biotech, Shanghai, China. Intracardiac injection of 50μL agomiR‐155 or miRNA scramble (0.5 nmol/g) was performed through the left ventricle after removing the suture knot to allow reperfusion. Tissues were collected for further detection 24 h later.

### 2.17 RNA sequencing

Total RNA was isolated using TRIzol reagent (15596–018, Invitrogen, CA, USA). The miRNA sequencing library was prepared and sequenced on Illumina HiSeq sequencer. The sequencing service was provided by CloudSeq Biotech (Shanghai, China). Detailed methods of RNA sequencing and data analysis were showed in supplementary material. The read statistics of miRNAs were listed in Table S1.

### 2.18 Real‐time quantitative polymerase chain reaction (RT‐qPCR)

Cells with indicated treatments and the apex of myocardia from mice in each group were used for RNA extraction with TRIzol regent (15596–018, Invitrogen, CA, USA). For mRNA, total RNA was transcribed into complementary DNAs (cDNAs) using the PrimeScript RT reagent kit (Takara Bio, Inc., Otsu, Japan). For miRNA, reverse transcription was performed using the microRNA Reverse Transcription Kit (Tiangen Biotech, Beijing, China). RT‐qPCR was performed in a 10 μL reaction system containing forward/reverse primers, cDNA, and SYBR Green MasterMix (Applied Biosystems; Thermo Fisher Scientific, Inc.) with three replicates. The miRNA and mRNA levels were respectively normalized to the endogenous control U6 small nuclear RNA and β‐actin. Relative expression of these RNAs was calculated using the 2^–∆∆CT^ method. All the primers used in the study were listed in Table S2.

### 2.19 ELISA

Cell culture supernatant and serum samples were collected and stored at ‐80˚C. Protein levels of IL1β (MultiSciences, China), IL6 (MultiSciences, China), TNFα (Absin Bioscience, China), Cxcl1 (MultiSciences, China) and Cxcl2 (MultiSciences, China) were determined with specific ELISA kits according to the manufacturer's directions.

### 2.20 Western blot

Protein concentrations were measured with the Bicinchoninic Acid (BCA) Protein Assay Kit (Bio‐Rad, Hercules, CA, USA). Equal volumes of protein samples were separated by sodium dodecylsulfate polyacrylamide gel electrophoresis and transferred to polyvinylidene difluoride (PVDF) membranes. The membranes were blocked with 5% non‐fat milk for 1 h at room temperature, and then incubated overnight at 4°C with antibodies specific for the CD63 (1:500 dilution, Abcam, UK), CD9 (1:1000 dilution, Abcam, UK), Alix (1:1000 dilution, Abcam, UK), or TSG101 (1:000 dilution, Abcam, UK), JAK2 (1:1000, Cell Signaling Technology, USA), p‐JAK2 (1:1000, Cell Signaling Technology, USA), STAT1 (1:1000, Cell Signaling Technology, USA), p‐STAT1 (1:1000, Cell Signaling Technology, USA), and β‐actin (1:5000, Santa Cruz Biotechnology, USA). On the following day, the membranes were washed and incubated with horseradish peroxidase (HRP)‐coupled secondary antibodies (1:1000, Cell Signaling Technology, USA). The blots were detected by enhanced chemiluminescence (Pierce, Rockford, Illinois) and quantified using the Bio‐Rad imaging system (Hercules, CA). β‐actin was used as a loading control and the relative intensity of the interested protein was normalized to that of the control group.

### 2.21 Statistical analysis

Data were shown as the mean ± Standard Error of Mean (SEM). Student's *t*‐test was applied for the comparison of two groups. Multiple groups comparison was performed using one‐way analysis of variance (ANOVA), followed by Tukey's multiple comparisons test. Correlation analysis was performed using Pearson's chi‐square test. GraphPad Prism 8.0 (GraphPad Software, San Diego, CA, USA) was used for all data analyses. *P* < 0.05 was considered statistically significant.

## 3 RESULTS

### 3.1 Cardiac EV alteration induced by IR

**FIGURE 1:**
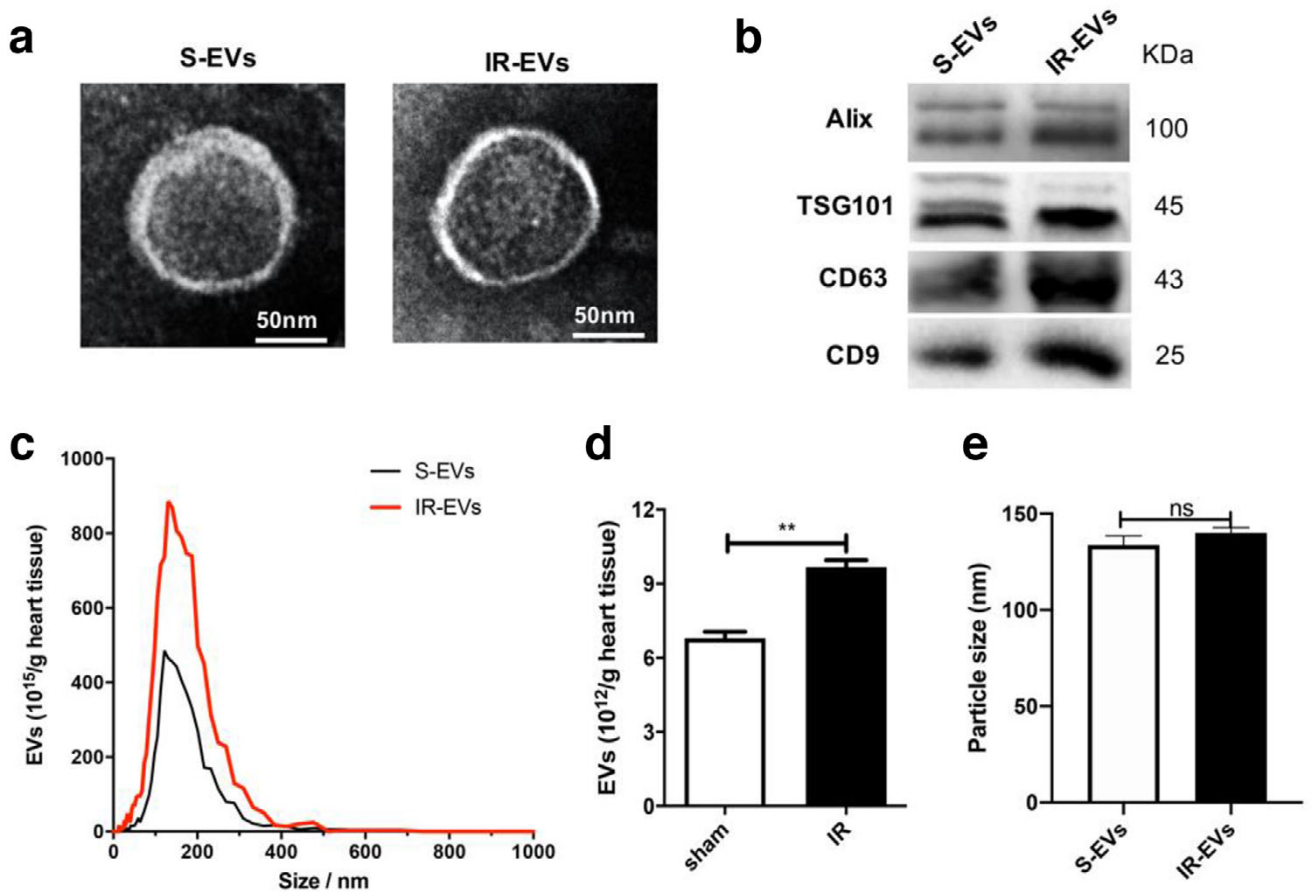
Characterization of cardiac EVs. [(a) A representative TEM image of sham (S‐EVs) and IR‐EVs, (bar = 50 nm). (b) Protein immunoblots of EVs, including four typical exosomal markers (Alix, Tsg101, CD63 and CD9). (c) Particle size distribution of cardiac EVs was measured using nanoparticle tracking analysis (NTA). (d) Quantification of EVs isolated from sham and IR injured hearts in (c). (e) EV size detected by NTA in (c). ^**^, *P* < 0.01; ns, not significant]

The typical morphology of EVs from sham or IR injured hearts were captured under TEM (Figure 1a). As shown in Figure 1b, typical exosome markers including CD63, CD9, Alix and TSG101 were expressed in sham (S‐EVs) and IR‐EVs. Myocardial IR significantly increased the intracardiac release of EVs (Figures 1c and d), but did not significantly modulate the size of the EVs (Figure 1e).

### 3.2 IR‐EVs aggravated IR induced heart injury and facilitated M1 polarization of macrophage in IR‐injured heart

**FIGURE 2:**
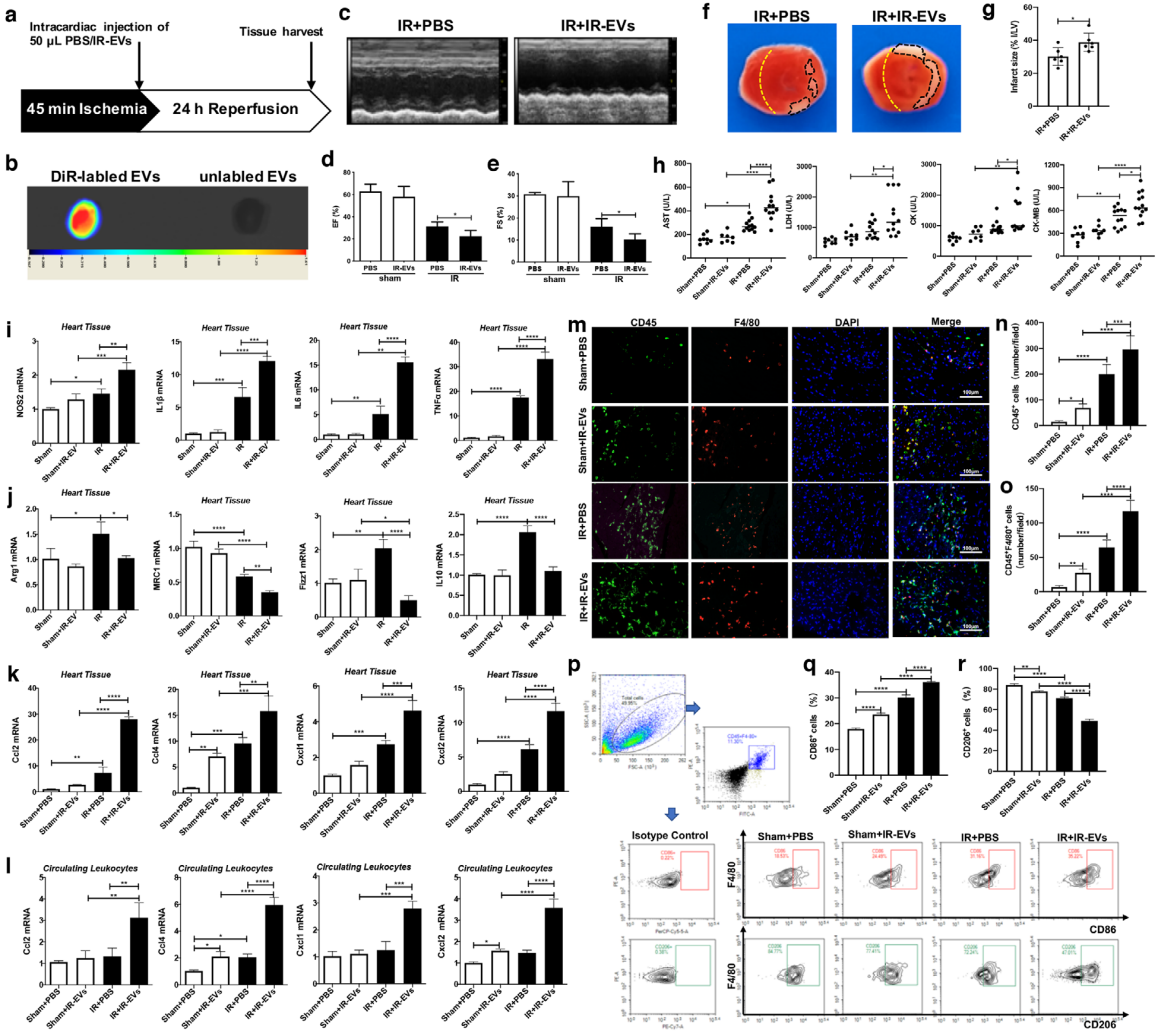
IR‐EVs transfusion aggravate IR injury and facilitate M1 polarization of macrophage in the heart. [(a) Protocol schematic for infusion of IR‐EVs and tissue harvest. (b) Bioluminescence imaging confirmed that IR‐EVs were retained within the heart by intracardiac injection of DiR‐labelled IR‐EVs. (c) Representative echocardiogram. Statistical results of (d) EF value, and (e) FS value (n = 6). (f) Representative TTC‐stained hearts from mice sacrificed 1 day after IR‐EVs or PBS transfusion. (g) Statistical results of the infarct size (represented with infarct area (I) / left ventricular (LV) area) in (f) (n = 6). (h) Plasma levels of myocardial enzymes, including AST, LDH, CK and CK‐MB (n = 8, 8, 12, 12). (i) The expression of NOS2, IL1β, IL6 and TNFα in the heart. (j) The expression of M2‐polarization related genes including Arg1, MRC1, Fizz1 and IL10 in the heart. The expression of chemokines including Ccl2, Ccl4, Cxcl1 and Cxcl2 in (k) the heart and (l) the circulating leukocytes. (m) Representative immunofluorescence images showed the CD45^+^ inflammatory cells and CD45^+^F4/80^+^ macrophages in the heart. (n) Statistical results of CD45^+^ inflammatory cells (cell counts per field) in the cardiac immunofluorescence images. (o) Statistical results of CD45^+^F4/80^+^ macrophages (cell counts per field) in the cardiac immunofluorescence images. (p) Polarization of macrophages in the heart determined by flow cytometry. (q) Percentage of CD86^+^ M1 polarized macrophage in the heart. (r) Percentage of CD206^+^ M2 polarized macrophage in the heart. ^*^, *P* < 0.05;^**^, *P* < 0.01; ^***^, *P* < 0.001; ^****^, *P*<0.0001]

Our previous study demonstrated an increased release of cardiac EVs post myocardial IR (Ge et al., 2019). To further clarify the role of IR‐EVs in cardiac IR injury, IR‐EVs were transfused into sham or IR‐injured heart (Figure 2a). Bioluminescence imaging showed that IR‐EVs can retain within the heart at 24 h after intracardiac injection of DiR‐labelled IR‐EVs (Figure 2b). More importantly, transfusion of IR‐EVs significantly impaired the heart function (Figure 2c‐e) and increased the infarct area (Figure 2f and g), compared with those receiving PBS vehicle. Compared with IR+PBS mice, mice in IR+IR‐EVs group displayed much higher levels of myocardial enzymes (Figure 2h). These results indicate that IR‐EVs aggravate IR induced heart injury.

Macrophage polarization plays vital roles in IR induced cardiac injury and repair. We next sought to determine whether IR‐EVs enhanced the inflammation and regulated the polarization of macrophages in vivo. Figure 2i showed that IR‐EV transfusion significantly enhanced the expression of M1‐polarization marker gene NOS2 and proinflammatory cytokines IL1β, IL6 and TNFα in IR‐injured hearts. While, mRNA levels of M2‐polarization related Arg1, MRC1, Fizz1 and IL10 decreased greatly (Figure 2j). Conspicuously, IR‐EVs transfusion enhanced the expression of chemokines including Ccl2, Ccl4, Cxcl1 and Cxcl2 in both the heart and the circulating leukocytes of IR‐injured mice (Figure 2k and l). IR‐EVs didn't enhance the expression of proinflammatory factors, such as IL1β and IL6 (Figure S1A and B), but did decrease the expression of IL10 in circulating leukocytes of IR‐injured mice (Figure S1C). Immunofluorescence staining of heart tissues revealed that IR‐EV transfusion promoted the infiltration of CD45^+^ inflammatory cells as well as CD45^+^F4/80^+^ macrophages in both the sham and IR‐injured mice (Figure 2m‐o). Previous study highlighted the role of Cxcl2 in promoting neutrophil infiltration during infection (Lentini et al., 2020). In addition to macrophages, we also observed increased recruitment of neutrophils in IR mice treated with IR‐EVs (Figure S2A‐C). Flow cytometry results further confirmed that IR‐EVs increased the proportion of CD86^+^ (M1 marker) macrophages and decreased the percentage of CD206^+^ (M2 marker) macrophages in both the sham and IR‐injured heart tissues (Figure 2p‐r). These results confirm that IR induced cardiac EVs intensify the local inflammation. Notably, IR‐EVs promote the infiltration of macrophages into the injured heart and contribute to their M1 polarization during cardiac IR.

### 3.3 Inhibition of EV production mitigated myocardial IR injury and attenuated local inflammation

**FIGURE 3:**
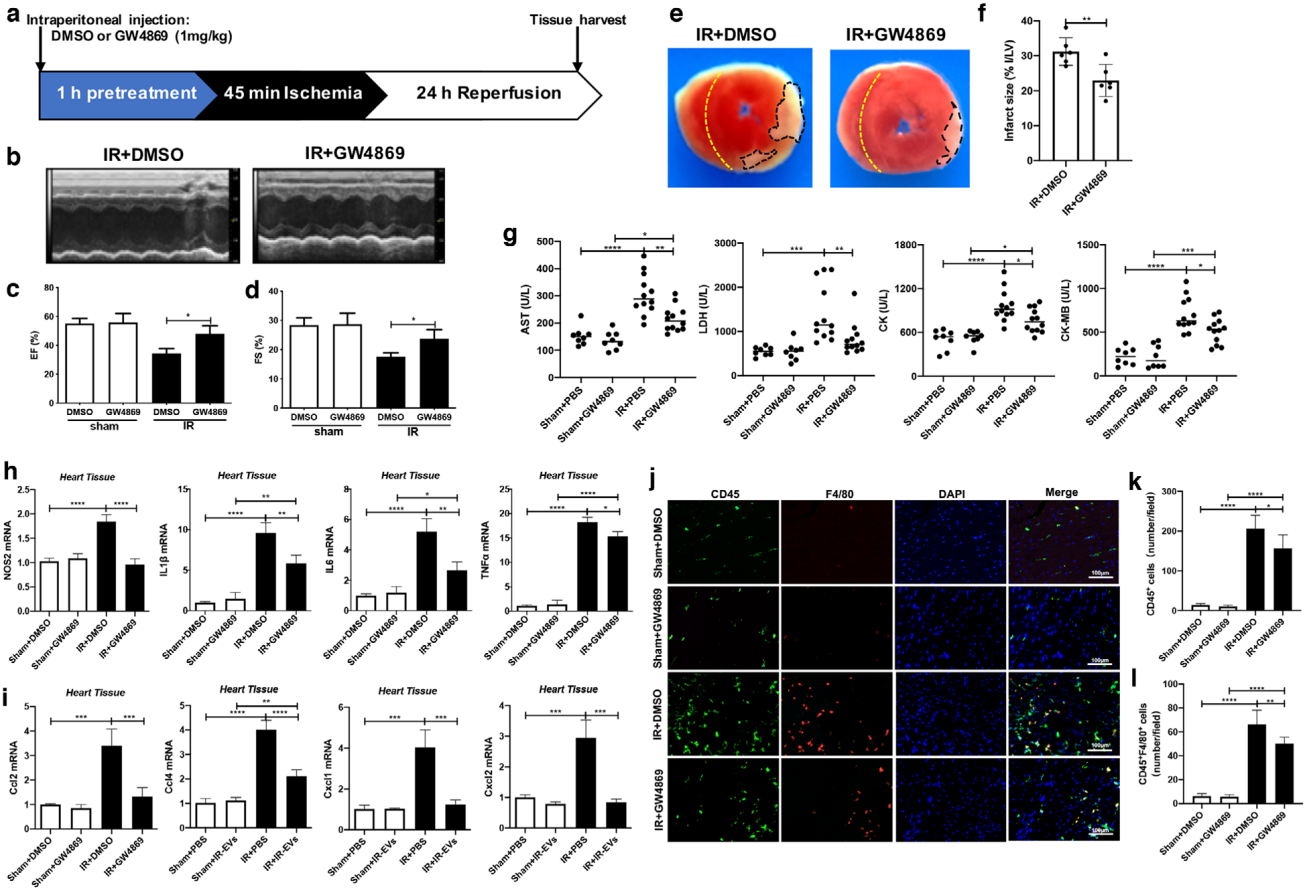
GW4869 treatment mitigates IR injury and attenuates local inflammation. [(a) Protocol schematic for GW4869 treatment and tissue harvest. (b) Representative echocardiography images. Statistical results of (c) EF value, and (d) FS value (n = 6). (e) Representative TTC‐stained hearts from GW4869 or DMSO treated mice 1 day post‐IR. (f) Statistical results of the infarct size (represented with infarct area (I) / left ventricular (LV) area) in (e) (n = 6). (g) Plasma levels of the myocardial enzyme, including AST, LDH, CK and CK‐MB (n = 8, 8, 12, 12). The expression of (h) M1‐polarization related genes including NOS2, IL1β, IL6 and TNFα, and (i) proinflammatory chemokines including Ccl2, Ccl4, Cxcl1 and Cxcl2 in the heart of IR mice treated with GW4869. (j) Representative immunofluorescence images of hearts stained with anti‐CD45 and anti‐F4/80 antibodies. (k) Statistical results of CD45^+^ inflammatory cells (cell counts per field) in the cardiac immunofluorescence images. (l) Statistical results of CD45^+^F4/80^+^ macrophages (cell counts per field) in the cardiac immunofluorescence images. These data were representative results (n = 3) of three repetitions. ^*^, *P* < 0.05;^**^, *P* < 0.01; ^***^, *P* < 0.001; ^****^, *P*<0.0001]

Next, we used GW4869, a widely used chemical inhibitor of EV/exosome biogenesis (Menck et al., 2017; Xiao et al., 2018) to explore the effect of EV suppression on IR induced cardiac injury (Figure 3a). First, we confirmed that pre‐treatment with GW4869 by intraperitoneal injection reduced the IR induced EV production in the heart (Supplemental Figure 3). Administration of GW4869 markedly improved the heart function of IR‐injured mice (Figure 3b‐d), reduced the infarct area (Figure 3e‐f) and decreased the levels of myocardial enzymes in IR mice (Figure 3g) compared with those receiving vehicle injection. In addition, we found GW4869 treatment significantly inhibited the expressions of NOS2, IL1β, IL6 and TNFα (Figure 3h), and upregulated the levels of Arg1, MRC1 and Fizz1 in IR injured hearts (Supplemental Figure 4A‐C). Similarly, compared with the IR mice, the expression of proinflammatory chemokines including Ccl2, Ccl4, Cxcl1 and Cxcl2 were diminished in the heart of IR mice treated with GW4869 (Figure 3i). Consistently, GW4869 treatment decreased the infiltration of CD45^+^ inflammatory cells as well as CD45^+^F4/80^+^ macrophages in the IR‐injured heart (Figure 3j‐l). The cardioprotective effects of IR‐EV inhibition by GW4869 further suggest that IR‐induced EVs are involved in the cardiac IR injury and contribute to the enhanced inflammation.

### 3.4 IR‐EVs programed macrophages towards M1‐like polarization in vitro

**FIGURE 4:**
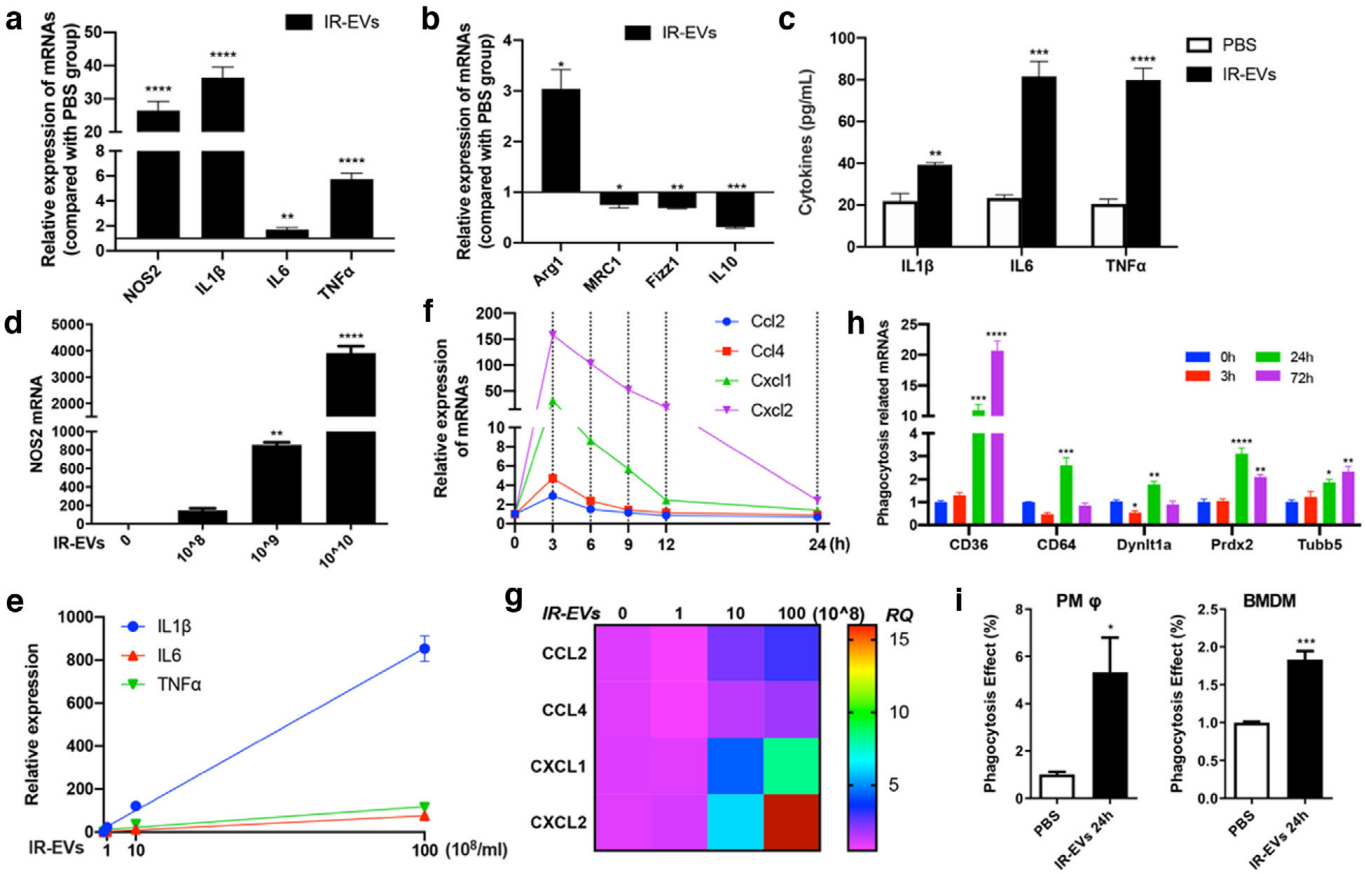
IR‐EVs facilitate M1‐like polarization of macrophages. [The expression of (a) M1 polarization related genes and (b) M2 polarization related genes in macrophages treated with IR‐EVs or PBS overnight. (c) The secretion of IL1β, IL6 and TNFα by macrophages treated with IR‐EVs or PBS overnight. (d) The expression of NOS2 in macrophages treated with gradient concentration (0, 10^8^, 10^9^ and 10^10^ /ml) of IR‐EVs overnight. (e) Positive correlations between IR‐EV concentration and the expression of proinflammatory factors in macrophages treated with IR‐EVs. (f) The expression of chemokines including Ccl2, Ccl4, Cxcl1 and Cxcl2 in macrophages treated with IR‐EVs for different times (0 h, 3 h, 6 h, 9 h, 12 h and 24 h). (g) The expression of chemokines including Ccl2, Ccl4, Cxcl1 and Cxcl2 in macrophages treated with gradient concentrations of IR‐EVs overnight. (h) The expression of phagocytosis related genes in macrophages treated with IR‐EVs for different times (0 h, 3 h, 24 h and 72 h). (i) Phagocytic function of peritoneal macrophages (PMφ) and bone marrow‐derived macrophages (BMDM) was detected by phagocytosis assay. These data were representative results (n = 3) of three repetitions. ^*^, *P* < 0.05;^**^, *P* < 0.01; ^***^, *P* < 0.001; ^****^, *P*<0.0001]

To further clarify the role of IR‐EVs on macrophages polarization, primary peritoneal macrophages were applied and co‐cultured with IR‐EVs. Firstly, addition of IR‐EVs promoted the expression of M1 polarization related genes, including NOS2, IL1β, IL6 and TNFα (Figure 4a and Figure S5A and C), and decreased the expression of M2 polarization related genes, like MRC1, Fizz1 and IL10 (Figure 4b and Figure S5B). Consistent results were also confirmed in the protein levels of classic proinflammatory factors including IL1β, IL6 and TNFα (Figure 4c). Continuously increased release of IL6 in cell culture media was found after IR‐EV stimulation for even 24 h (Figure S5D). Moreover, the mRNA level of NOS2 (Figure 4d), IL1β, IL6 and TNFα (Figure 4e) upregulated gradually as the concentration of EVs increased in the cell culture system. Further, we explored the effects of IR‐EVs on the production of chemokines in vitro. Chemokines including Ccl2, Ccl4, Cxcl1 and Cxcl2 were all significantly increased at the mRNA level, especially Cxcl2 (Figure 4f and Figure S6). Compared with the untreated group, high‐level IR‐EVs (> 10^9^ /ml) resulted in a high expression of chemokines, especially Cxcl2 (Figure 4g). Our in vivo and in vitro results suggest that the release of a large number of IR‐EVs after IR may be one of the driving factors that promote the production of chemokines and inflammatory factors. In addition, treatment with IR‐EVs significantly elevated the expression of phagocytic‐related genes (Figure 4h), and promoted the phagocytosis activity as well in both PMφ and BMDMs (Figure 4i).

### 3.5 miR‐155‐5p as a candidate effector of IR‐EV‐mediated macrophage polarization

**FIGURE 5:**
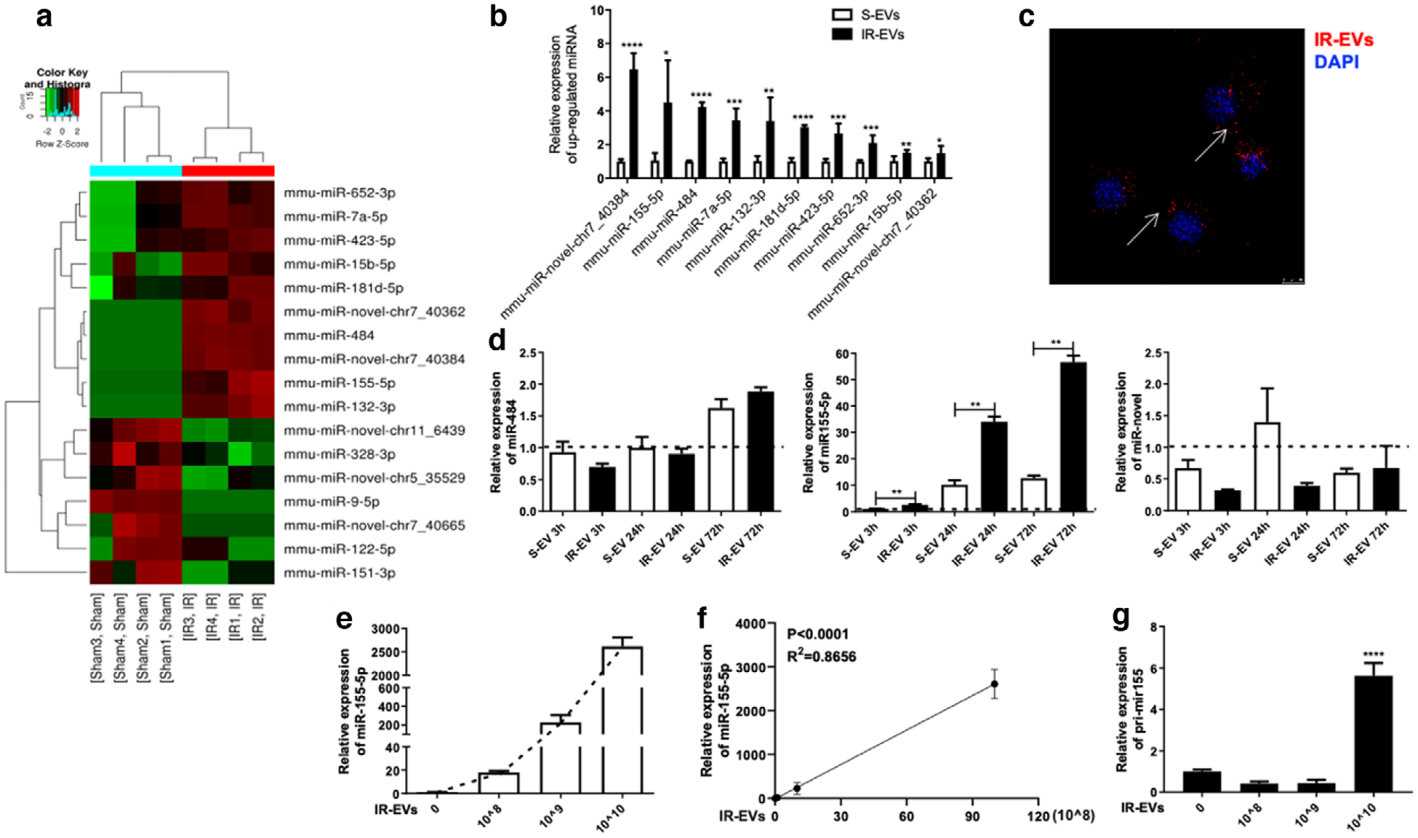
MiR‐155‐5p can be transferred from IR‐EVs to macrophages. [(a) Heat map showing the differential expressed miRNAs between S‐EVs and IR‐EVs. (b) The significantly high‐expressed miRNAs in IR‐EVs were verified by qPCR. (c) Confocal images showing the uptake of IR‐EVs by PMφ after coculture with DiI‐labelled EVs for 24 h. (d) qPCR was used to assess the expression of 3 IR‐EV–enriched miRNAs, including miR‐484, miR‐155‐5p and a novel miRNA (miR‐novel) in PMφ treated with PBS, S‐EVs or IR‐EVs for different times (0 h, 3 h, 24 h and 72 h). (e) Addition of increasing concentrations of IR‐EVs results in a dose‐dependent increase in miR‐155‐5p in treated macrophages. (f) A positive correlation between IR‐EV concentration and the expression of miR‐155‐5p in IR‐EV–treated macrophages. (g) The expression of pri‐mir155 in macrophages treated with gradient concentrations of IR‐EVs for 24 h. These data were representative results (n = 3) of three repetitions. ^*^, *P* < 0.05;^**^, *P* < 0.01; ^***^, *P* < 0.001; ^****^, *P*<0.0001]

EVs are known as cell‐to‐cell communication vehicles by delivering miRNAs. We therefore performed RNA‐seq of EVs from sham (S‐EVs) and IR‐injured heart to identify the candidate miRNA effector. Differential expressed miRNAs were identified (Figure 5a and Table S3), and the top 10 high‐expressed miRNAs in IR‐EVs were also verified by qPCR, including miR‐155‐5p, miR‐484, miR‐7a‐5p, miR‐132‐3p, miR‐181d‐5p, miR‐432‐5p, miR‐652‐3p, miR‐15b‐5p and 2 novel miRNAs: miR‐novel‐chr7_40384 and miR‐novel‐chr7_40362 (Figure 5b). Results from confocal microscopy revealed that IR‐EVs can be uptaken by macrophages, which consistently embodied the carrier property (Figure 5c). To further determine the candidate miRNA of IR‐EV‐mediated macrophage polarization, the top three high‐expressed miRNAs (miR‐155‐5p, miR‐484 and miR‐novelR‐chr7_40384) were tested in PMφ cocultured with IR‐EVs. Remarkably, miR‐155‐5p was significantly up‐regulated in IR‐EV cultured macrophages compared with S‐EV cultured ones (Figure 5d). Moreover, the concentration of IR‐EVs was positively correlated with the expression of miR‐155‐5p in the cocultured macrophages (Figure 5e and f). Interestingly, high concentration of IR‐EVs (10^10^/ml) increased the expression of pri‐mir155 at the same time (Figure 5g). Consistently, we further found highly enriched miR‐155‐5p in circulating IR‐EVs compared with the circulating sham‐EVs (Supplemental Figure 7a). The low‐expressed miRNAs, such as miR‐9‐5p and miR‐151‐3p in cardiac IR‐EVs were also decreased in the circulating IR‐EVs (Figure S7B and C). Therefore, miR‐155‐5p may act as a strong candidate for the key regulatory cargo contained in IR‐EVs.

### 3.6 miR‐155‐5p in IR‐EVs promoted macrophage M1 polarization through JAK2/STAT1 pathway

**FIGURE 6:**
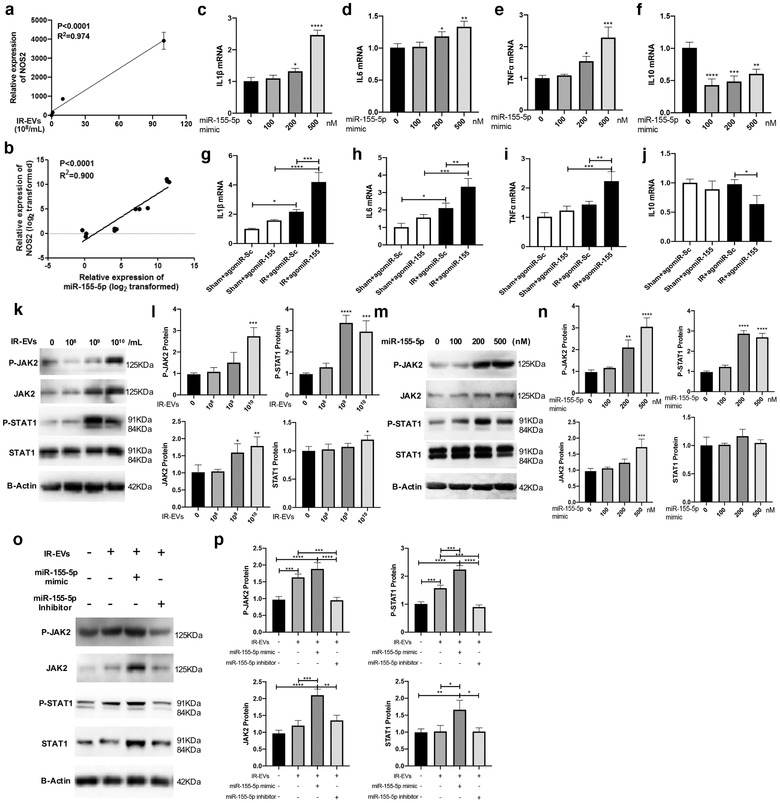
miR‐155‐5p facilitate M1 polarization by activating JAK2/STAT1 pathway. [(a) A positive correlation between IR‐EV concentration and NOS2 expression in IR‐EV–treated macrophages. (b) A positive correlation between the expression of miR‐155‐5p and NOS2 expression in IR‐EV–treated macrophages. The expression of proinflammatory factors (c) IL1β, (d) IL6, (e) TNFα and (f) IL10 in PMφ treated with miR‐155‐5p mimics. In vivo agomiR‐155 administration increased the expression of proinflammatory cytokines (g) IL1β, (h) IL6, (i) TNFα, and decreased the expression of the anti‐inflammatory cytokine (j) IL10 in IR‐injured hearts. (k) Western blotting confirms that JAK2 and STAT1 protein is activated by IR‐EV (0, 10^8^, 10^9^ and 10^10^ /ml) treatment. (l) Quantified data of immunoblotting band intensity in (k). (m) MiR‐155‐5p (0, 100, 200, 500 nM) overexpression in macrophages activates JAK2 and STAT1 protein as assessed by western blot. (n) Quantified data of immunoblotting band intensity in (m). (o) Western blotting confirms that miR‐155‐5p mimic further enhances IR‐EVs induced JAK2/STAT1 activation, and miR‐155‐5p inhibitor counteracts the elevation of p‐JAK2 and p‐STAT1 protein induced by IR‐EVs in macrophages. (p) Quantified data of immunoblotting band intensity in (o). These data were representative results (n = 3) of three repetitions. ^*^, *P* < 0.05;^**^, *P* < 0.01; ^***^, *P* < 0.001; ^****^, *P*<0.0001]

In addition to the significant increased NOS2 expression in PMφ cocultured with IR‐EVs, we further confirmed a positive association between IR‐EV concentration and NOS2 expression (Figure 6a). Meanwhile, miR‐155‐5p expression was positively correlated with NOS2 expression in IR‐EV–cocultured macrophages (Figure 6b). Transfection of miR‐155‐5p mimics increased the expression of proinflammatory factors (Figure 6c‐e) and decreased the expression of an anti‐inflammatory cytokine IL10 (Figure 6f). Moreover, intracardiac administration of agomiR‐155 reproduced the proinflammatory effect of IR‐EVs with increased expression of IL1β, IL6 and TNFα (Figure 6g‐i), and decreased expression of IL10 (Figure 6j) in IR‐injured heart. Previous studies indicated that JAK/STAT1 was an important regulatory pathway for M1 polarization of macrophages (Jin et al., 2020; Melbourne et al., 2020). As shown by Figure 6k and l, IR‐EVs significantly elevated the expression of total JAK2 and phosphorylated JAK2 (p‐JAK2) in PMφ. The downstream molecule STAT1 was also activated in IR‐EV–cocultured macrophages. Similarly, addition of miR‐155‐5p mimics activated the JAK2/STAT1 pathway in macrophages (Figure 6m and n). Additionally, miR‐155‐5p mimics further enhanced IR‐EVs induced JAK2/STAT1 activation, and miR‐155‐5p inhibitor counteracted the elevation of p‐JAK2 and p‐STAT1 protein induced by IR‐EVs in macrophages (Figure 6o and p). These results demonstrated that miR‐155‐5p contributed to IR‐EV–regulated M1 polarization of macrophages through JAK2/STAT1 activation.

### 3.7 IR‐EVs contributed to systemic inflammation in distant organs

**FIGURE 7:**
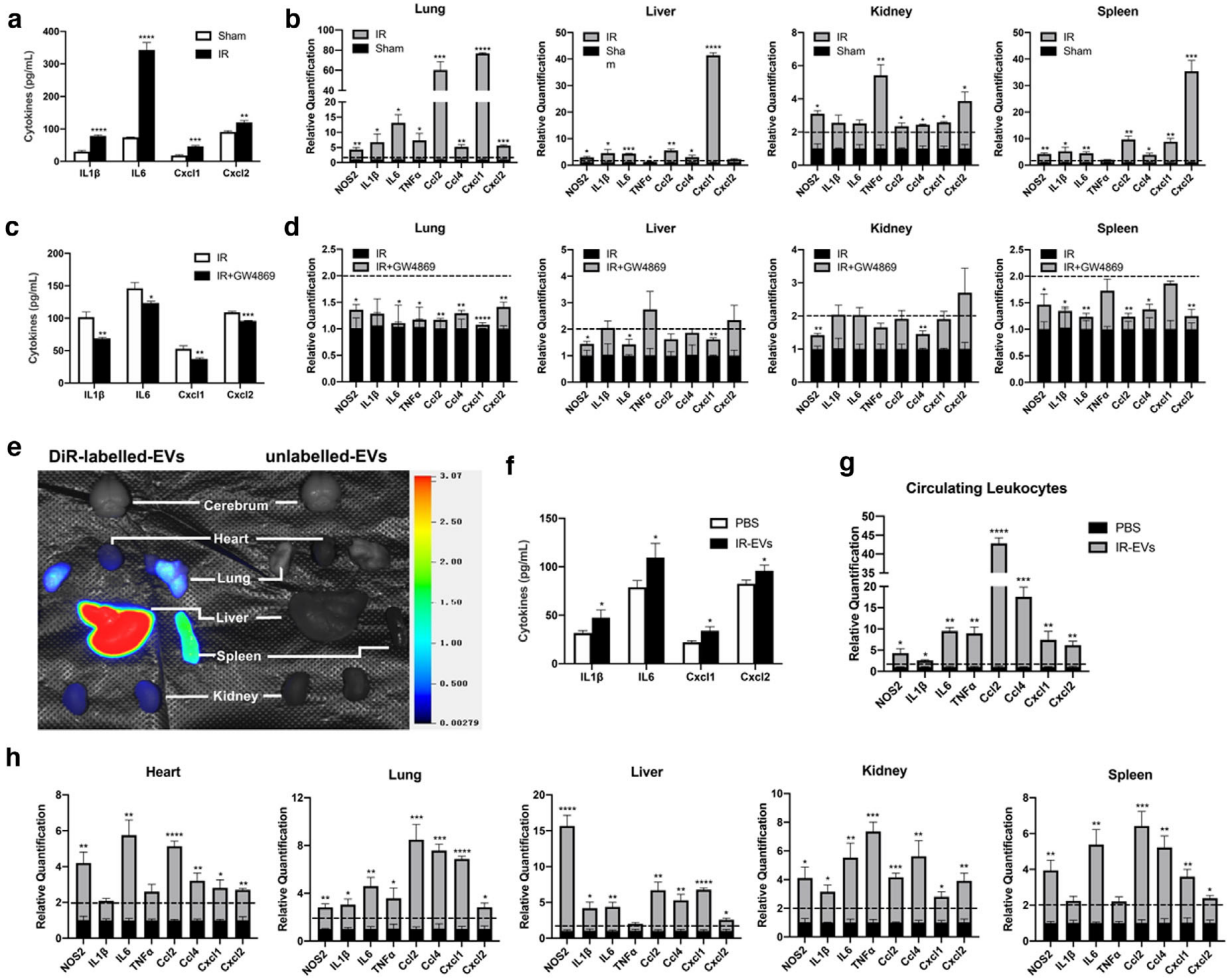
IR‐EVs trigger systemic inflammation in the distant organs. [(a) Myocardial IR injury promoted the secretion of proinflammatory cytokines (IL1β and IL6) and chemokines (Cxcl1 and Cxcl2) in the blood. (b) Myocardial IR injury induced increased expressions of proinflammatory genes in the distant organs. (c) GW4869 treatment reduced the release of proinflammatory cytokines (IL1β and IL6) and chemokines (Cxcl1 and Cxcl2) in the circulation of IR‐injured mice. (d) GW4869 treatment decreased the expressions of proinflammatory genes induced by myocardial IR in the distant organs. (e) Bioluminescence imaging showed the distribution of IR‐EVs among the organs 24 h after intravenous injection of DiR‐labelled EVs. (f) The secretion of proinflammatory cytokines (IL1β and IL6) and chemokines (Cxcl1 and Cxcl2) in the blood 24 h after intravenous injection of 100 μL IR‐EVs (0.4 μg/μL) or PBS. (g) The expression of proinflammatory genes in the circulating leukocytes 24 h after intravenous injection of 100 μL IR‐EVs (0.4 μg/μL) or PBS. (h) The expression of proinflammatory genes in different organs 24 h after intravenous injection of 100 μL IR‐EVs (0.4 μg/μL) or PBS. These data were representative results (n = 3) of three repetitions. ^*^, *P* < 0.05;^**^, *P* < 0.01; ^***^, *P* < 0.001; ^****^, *P*<0.0001]

To further explore whether IR‐EVs exert their roles in other organs, we firstly confirmed that myocardial IR injury triggered the increased release of EVs in the circulation (Figure S8A). Moreover, IR injury fostered the secretion of proinflammatory cytokines (IL1β and IL6) and chemokines (Cxcl1 and Cxcl2) in the circulation 1 day post IR (Figure 7a). In addition to heart tissues, increased expressions of proinflammatory genes were also presented in the distant organs including lung, liver, kidney and spleen (Figure 7b). Moreover, GW4869 treatment reduced the release of proinflammatory cytokines (IL1β and IL6) and chemokines (Cxcl1 and Cxcl2) in the circulation of IR‐injured mice (Figure 7c) and decreased the expressions of proinflammatory genes induced by myocardial IR in the distant organs (Figure 7d). As to brain tissues, cardiac IR injury only posed a feeble proinflammatory effect (Figure S8B), and we didn't find significant anti‐inflammatory effect of GW4869 treatment in the brain of IR‐injured mice (Figure S8C).

Recent studies highlighted EVs as distal regulatory molecules in different pathological processes (Thomou et al., 2017; Whitham et al., 2018). Bioluminescence imaging confirmed that IR‐EVs resided in various organs after intravenous injection of DiR‐labelled EVs to normal mice for 24 h (Figure 7e and Figure S8d). Next, we demonstrated that intravenous administration of IR‐EVs increased the release of proinflammatory cytokines (IL1β and IL6) and chemokines (Cxcl1 and Cxcl2) in the circulation (Figure 7f). The expression of proinflammatory genes in the circulating leukocytes were also elevated after intravenous injection of IR‐EVs (Figure 7g). Consistently, similar proinflammatory effects by intravenous administration of IR‐EVs were found in various organs (Figure 7h and Figure S8E). Moreover, administration of IR‐EVs upregulated the expression of CD45+ and CD45+F4/80+ cells in multiple organs especially in EV‐enriched ones, such as lung and liver (Figure S9). These findings suggest that IR‐EVs promote a systemic inflammatory response during cardiac IR injury.

## 4 DISCUSSION

Increasing studies highlight the importance of inflammation in the heart injury and healing. It is also becoming clearer that macrophages heterogeneity (typically defined by M1 and M2 states) is a nuanced process highly depending on the microenvironmental cues including EVs (Ismail et al., 2013). The present study for the first time disclosed the miRNA contents in cardiac EVs and demonstrates the vital role of IR‐EVs in promoting the proinflammatory, chemotactic and phagocytic function of macrophages and aggravating IR induced cardiac injury and dysfunction. Importantly, we confirm that EV inhibition by GW4869 mitigates IR‐induced cardiac inflammation and injury. We further reveal that IR‐EVs transfer its cargo miR‐155‐5p to macrophages and promote the proinflammatory M1 polarization via activating JAK2/STAT1 pathway. Moreover, IR‐EVs boost not only the local inflammation in the heart but also create a proinflammatory environment in the distant organs during myocardial IR injury. Therapeutic targeting of the proinflammatory EVs may be a promising approach to control the inflammation and protect the heart from IR injury.

EVs carry biological information from their mother cells. Most of the previous investigations focused on the role of EVs/exosomes isolated from culture medium of different cells, including cardiomyocytes (Wang et al., 2020) and fibroblasts (Bang et al., 2014). However, the EVs derived from cell models can't represent the real situation of the body. In the present study, we applied the classic in‐situ IR injury murine model by ligating the LAD coronary artery for 45 min before reperfusion. We disclosed the deleterious effect of IR‐EVs on cardiac injury by both adoptive IR‐EV transfusion and treatment with GW4869 as an inhibitor of EV generation and release. A previous report demonstrated that cardiac ischemic preconditioning, where the isolated donor heart is submitted to brief cycles (3×5‐5 min) of ischemia‐reperfusion, protect the recipient heart from cardiac infarction (Giricz et al., 2014). These seemingly contradictory results further revealed the close association between the function/contents of EVs and the state of the source cells and organs. Inspiringly, we found that a single injection of GW4869 was able to reduce IR induced heart injury. Therefore, a timely inhibition of the detrimental EVs should be a promising strategy for IR injury. Another study indicated that GW4869 can inhibit N‐SMase activation and protect the TNF‐treated cells from death (Luberto et al., 2002). Thus, GW4869 may also exert its roles via other mechanism.

Inflammation and heart diseases are strongly connected and mutually reinforce each other. Ischemic heart injury provokes sterile inflammation in the heart itself. Long‐term or excessive inflammatory response aggravates heart injury after acute MI (Eskandari et al., 2018; Fang et al., 2017). This points out the need to control the inflammatory process at an early stage to avoid the persistent inflammation and heart injury. Release of damage associated molecular patterns (DAMPs) by stressed, malfunctioning, or necrotic cells during IR is widely known to provoke sterile inflammation and recruitment of immune cells (Liu et al., 2017; Turner, 2016). We newly find that IR‐EVs boost the inflammation and infiltration of immune cells (especially macrophages and neutrophils demonstrated in our study) in the infarct heart, which supports a role for IR‐EVs in the initiation of inflammatory phase in the heart following IR. Furthermore, GW4869 treatment dampened the inflammatory injury at the early stage with reduced pro‐inflammatory cytokines. As such, we proposed that the detrimental effect of IR‐EVs potentially attributed to the excessive inflammation induced by accumulated and activated inflammatory cells. Inhibition of the proinflammatory EVs during IR helps break the inflammation‐injury circle and eventually alleviate the cardiac injury. In addition to local inflammation induced by direct cardiac injury, it is still a mystery that heart disease is usually accompanied by a systemic low‐grade inflammation (Scally et al., 2019; Yuan et al., 2019). As shown in our study, IR injury not only increased the inflammation in the heart but also in distant organs, and GW4869 treatment dampened the systemic inflammation in IR mice. These findings enlightened us on the link between IR‐EVs and the systemic inflammation during IR. The proinflammatory effect in healthy mice injected with IR‐EVs directly revealed that IR‐EVs as inflammatory mediators could initiate a systemic inflammation and increase the infiltration of immune cells in multiple organs. Previous study revealed that injection of exosomes isolated from blood of AMI mice into wild‐type mice can downregulate CXCR4 expression in bone marrow mononuclear cells and increase the number of circulating progenitor cells (Cheng et al., 2019). These evidences suggested that, in addition to local injured heart, cardiac IR‐EVs also enter the circulation, as well as other tissues and organs to recruit inflammatory cells into remote organs. Our in vitro study indicated that IR‐EVs can directly influence the inflammatory state of macrophages. Thus, these IR‐EVs may also directly interact with the cells (such as macrophages) in remote organs to induce inflammatory response. It is also possible that EVs induced by other stress or injury are also involved in initiating and sustaining the inflammatory state in the body.

EVs exert their regulative functions on various immune cells including T cells (Okoye et al., 2014), B cells (Carreras‐Planella et al., 2019), monocyte/macrophages (L et al., 2019) and dendritic cells (Shahir et al., 2020). As a major population of immune cells in heart tissue, macrophages play an important role in the development of heart injury through M1/M2 polarization (Cao et al., 2018; Fan et al., 2018). Different origins of EVs have divergent roles on macrophage polarization. Hepatocyte EVs induced by lipids can activate an inflammatory phenotype in macrophages (Hirsova et al., 2016). While, mesenchymal stem cell‐derived EVs exert anti‐inflammatory polarization (Lo Sicco et al., 2017). Our in vivo and in vitro results revealed a significant proinflammatory effect of IR‐EVs and further confirmed that IR‐EVs contributed to the M1 macrophages mediated inflammatory responses. Tissue macrophages can synthesize neutrophil chemoattractant CXCL1/CXCL2 in response to pathological stimulus (De Filippo et al., 2013). Remarkably, we found significantly high expression of Cxcl2 in macrophages induced by IR‐EVs. Moreover, in vivo IR‐EV administration increased the expression of Cxcl2 and the recruitment of neutrophils in IR injured heart. In this process, macrophages may act as a bridge. However, whether IR‐EVs act directly on neutrophils resulting in their accretion in the heart need further exploration. It is reported that high levels of Cxcl2 in the heart tissue aggravated myocardial infarction, and blocking Cxcl2 and its receptors reduced the infarct sizes (Mylonas et al., 2017). Other studies revealed that Cxcl2 can promote neutrophil infiltration (Lentini et al., 2020) and activate NLRP3 inflammasome (Boro & Balaji, 2017), which is essential for excessive inflammatory damage during IR injury (Frangogiannis, 2006). Therefore, IR‐EVs deteriorate the IR injury by triggering the M1 polarization in the heart and Cxcl2 may be a key mediate in this process, which further promote the neutrophil infiltration and magnify the inflammatory responses.

MicroRNAs regulate gene expression and cell processes. We firstly identified that miR‐155‐5p was enriched in IR‐EVs, and studies have demonstrated the involvement of miR‐155‐5p in inflammatory response regulation (Wang et al., 2019; Zhang et al., 2019). High expression of miRNA‐155 is confirmed in M1 polarized macrophages (Essandoh et al., 2016, Lu et al., 2016). We further verified that miR‐155‐5p facilitated macrophages to M1 polarization. Addition of IR‐EVs displayed the similar effect on macrophages by transferring its cargo miR‐155‐5p. Moreover, the primary miR‐155 (pri‐mir155) expression was also increased when macrophages were treated with high concentration of IR‐EVs. It is likely that proteins or lipids contained in IR‐EVs also contribute to the proinflammatory effects by directly promoting miR‐155‐5p expression. In macrophages, the positive correlation between the stimulus concentration of IR‐EVs and miR‐155‐5p expression and between the expression of miR‐155‐5p and M1 polarization marker gene NOS2 further clarified the IR‐EVs**–**miR‐155‐5p**–**M1 polarization axis in the heart during IR injury. Further in vivo experiments to determine the characteristics of intracardiac monocytes after injection or reversion of the healing phenotype using an inhibitor will strengthen the importance of miRNA‐155 in IR‐EVs in cardiac IR injury.

Previous studies highlight that JAK‐STAT1 is an important pathway to regulate macrophage M1 polarization (Jin et al., 2020; Melbourne et al., 2020). MiR‐155‐5p was reported to inhibit SOCS1 expression and activate JAK‐STAT1 pathway (Wang et al., 2018). In rheumatic heart disease, miR‐155 aggravated the valve injury through S1PR1, SOCS1/STAT3 and IL‐6/STAT3 signalling pathways (Chen et al., 2020). In cardiac IR injury, we confirmed that IR‐EVs enhanced M1 polarization of macrophages through miR‐155‐5p induced JAK2‐STAT1 activation. Exosomal miRNA‐155‐5p can promote macrophage infiltration in tumour tissues (Feng et al., 2019). In our study, IR‐EVs increased macrophage infiltration in the heart, and miRNA‐155‐5p in IR‐EVs is probably the potential effector. In addition to JAK/STAT1, we found that IR‐EVs influenced multiple inflammatory pathways. In macrophages, IR‐EVs upregulated the mRNA levels of proinflammatory STAT1 and STAT3, and downregulated the expression of anti‐inflammatory PPARγ (Figure S10 A‐C and F). High level of IR‐EVs (> 10^9^/ml) also increased the mRNA expression of anti‐inflammatory SOCS1 and SOCS3 (Figure S10D and E), which may attribute to a negative feedback regulation induced by the excessive inflammation.

In addition, the miRNA sequencing analysis revealed low expression of some anti‐inflammatory miRNAs, such as miR‐9‐5p (Ou et al., 2019) and miR‐151‐3p (Liu et al., 2018) in IR‐EVs compared with S‐EVs, which possibly further aggravated the imbalance of inflammation and exacerbated inflammatory injury in the heart post IR. In the study, GO analysis showed that the differentially expressed miRNAs may mainly affect the process of cell metabolism (Figure S11 and 12). Previous studies have also confirmed that miRNAs regulate mitochondrial function and affect energy metabolism in IR injury (Hu et al., 2015). Therefore, cell metabolism is probably another important target of IR‐EVs. Further studies are expected to reveal the other novel mechanisms of IR injury mediated by these EVs. Moreover, cardiac EVs are released by different cells, such as cardiomyocytes, endothelial cells and fibroblasts. We found an increased release of cardiomyocyte‐derived (CX43^+^) and endothelium‐derived (CD106^+^) EVs in the IR‐injured heart (data not shown). How EVs with different origin exert their functions during IR injury need further exploration.

**FIGURE 8:**
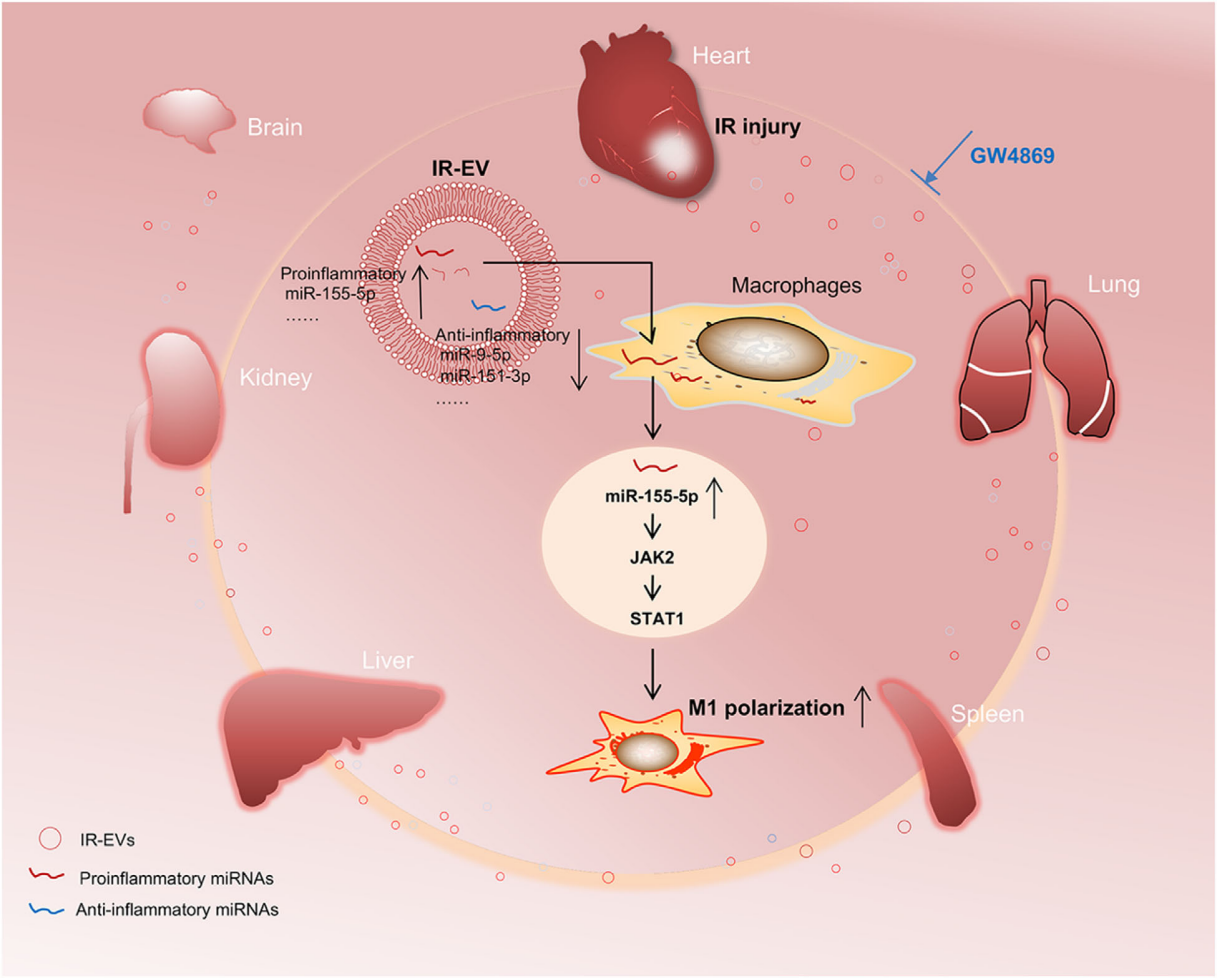
Schematic diagram of the role of IR induced EVs in myocardial IR injury. [Myocardial IR elicit a massive release of cardiac EVs. These IR‐induced EVs (IR‐EVs) boost the local sterile inflammation in the IR‐injured heart and can be shunted to the distant organs via circulation initiating systemic inflammation. Markedly, IR‐EVs can deliver miR‐155‐5p into macrophages and promote a proinflammatory phenotype through activating the JAK2/STAT1 pathway. EV inhibition by GW4869 declines the local and systemic inflammation and significantly mitigates IR induced heart injury.]

In summary, the present study newly discloses that cardiac IR induced EVs aggravate IR injury and contribute to both the local and systemic inflammation. EV inhibition by GW4869 protects the heart from IR injury. Specially, we disclose the miRNA profile in cardiac EVs, and identify an IR‐EVs**–**miR‐155‐5p**–**M1 polarization axis in the heart during IR (Figure 8). These findings broaden our understanding of IR pathological process and shed new lights on the role of cardiac EVs in the injury, supporting the therapeutic potential by targeting IR‐EVs to alleviate IR injury and improve cardiac function in clinical settings.

## SOURCES OF FUNDING

The present study was supported by the National Nature Science Foundation of China (NSFC; grant nos. 81670458, 81470393 and 81370434), Shanghai Municipal Health and Family Planning Commission [grant nos. ZY(2018‑2020)‑FWTX‑2007], The Science and Technology Commission of Shanghai Municipality (grant nos. 17431906600) and The Top‐level Clinical Discipline Project of Shanghai Pudong (PWYgf2018‐05) .

## DISCLOSURES

None.

5

## Supporting information

Supporting information.

## Data Availability

The data supporting the findings of this study are available within the article and its supplementary materials.
