# Supplementary material for: Myocardial ischemia‐reperfusion induced cardiac extracellular vesicles harbour proinflammatory features and aggravate heart injury
Source: J Extracell Vesicles. 2021 Feb 23;10(4):e12072. doi: 10.1002/jev2.12072 (PMC7902529; doi:10.1002/jev2.12072)
Supplement: Supplementary file 1 — Supporting information. [file JEV2-10-e12072-s001.docx]

Supplementary Material

**Myocardial Ischemia-Reperfusion Induced Cardiac Extracellular Vesicles Harbor Proinflammatory Features and Aggravate Heart Injury**

Xinyu Ge^1-4^, Qingshu Meng^1-3^, Lu Wei^1-3^, Jing Liu^1-4^, Mimi Li^1-3^, Xiaoting Liang^1-3^, Fang Lin^1-3^, Yinzhen Li^1,6^, Yuhui Zhang^7^, Zhongmin Liu^1-5^, Huimin Fan ^2-5*^, Xiaohui Zhou^1-3*^

1. Research Center for Translational Medicine, Shanghai East Hospital, Tongji University School of Medicine, Shanghai 200120, P.R. China;
2. Shanghai Heart Failure Research Center, Shanghai East Hospital, Tongji University School of Medicine, Shanghai 200120, P.R. China;
3. Institute of Integrated Traditional Chinese and Western Medicine for Cardiovascular Chronic Diseases, Tongji University School of Medicine, Shanghai 200120, P.R. China；
4. Department of Cardiovascular Surgery, Shanghai East Hospital, Tongji University School of Medicine, Shanghai 200120, P.R. China;
5. Department of Heart Failure, Shanghai East Hospital, Tongji University School of Medicine, Shanghai 200120, P.R. China;
6. Department of Respiratory Medicine, Shanghai East Hospital, Tongji University, School of Medicine, Shanghai 200120, P.R. China;
7. Department of Ultrasound, Shanghai East Hospital, Tongji University, School of Medicine, Shanghai 200120, P.R. China.

^*^ Address for Correspondence:

*Xiaohui Zhou, MD, PhD*

Shanghai East Hospital, Tongji University School of Medicine, No. 150 Jimo Rd., Shanghai 200120, P.R. China. Tel: 0086-2161569884; Fax: 0086-2158798999; Email: zxh100@tongji.edu.cn

*Huimin Fan, MD, PhD*

Shanghai East Hospital, Tongji University School of Medicine, No. 150 Jimo Rd., Shanghai 200120, P.R. China. Tel: 0086-2161569884; Fax: 0086-2158798999; Email: frankfan@tongji.edu.cn.

**Supplemental Methods**

**RNA sequencing and data analysis**

RNA from EVs was extracted using TRIzol regent (15596–018, Invitrogen, CA, USA). RNA concentration was detected by NanoDrop. The quality of RNA library was tested by Agilent 2100 Bioanalyzer. High throughput sequencing service was provided by CloudSeq Biotech (Shanghai, China). Briefly, total RNA was used to prepare the miRNA sequencing library. The libraries were denatured as single-stranded DNA molecules, captured on Illumina flow cells, amplified in situ as clusters and sequenced on Illumina HiSeq sequencer following the manufacturer's instructions.

Data were generated after sequencing by Illumina HiSeq sequencer. Firstly, Q30 was used to perform quality control. The adaptor sequences were trimmed and the adaptor-trimmed-reads (≥15nt) were left by Cutadapt software (v1.9.3). Then, trimmed reads from all samples were pooled, and miRDeep2 software (v2.0.0.5) was used to predict novel miRNAs. The trimmed reads were aligned to the merged pre-miRNA databases (known pre-miRNA from miRBase plus the newly predicted pre-miRNAs) using Novoalign software (v3.02.12) with at most one mismatch. The numbers of mature miRNA mapped tags were defined as the raw expression levels. The read counts were normalized by TPM (tag counts per million aligned miRNAs) approach. Differentially expressed miRNA between two groups were filtered by Fold Change and P-value based on volcano plot. The target gene of each miRNA were predicted by miRNA target prediction tools including TargetScan and miRanda. The miRNA-targets networks were plotted by Cytoscape software (v2.8.0), and the Gene Ontology (GO) and KEGG pathway analysis were performed based on the top 10 differentially expressed miRNA target genes.

**Isolation and quantification of circulating EVs**

Circulating EVs in the plasma were isolated using ExoQuickTM Exosome precipitation solution (System Biosciences, USA) and the concentration was determined using the EXOCET ELISA assay (System Biosciences, USA) according to the manufacturer’s instructions.

**Supplemental** **Tables**

**Supplemental** **Table I.** The read statistics of miRNAs.

| **Sample Name** | **Raw reads** | **Adaptor Trimmed Reads (>=15nt)** | **Aligned Reads** |
| --- | --- | --- | --- |
| sham1 | 11,271,291 | 9,196,267 | 370,747 |
| sham2 | 11,705,327 | 8,639,103 | 358,057 |
| sham3 | 11,155,542 | 9,288,021 | 531,265 |
| sham4 | 11,684,981 | 8,575,351 | 469,532 |
| IR1 | 10,730,946 | 9,642,428 | 414,783 |
| IR2 | 9,905,490 | 8,900,881 | 383,521 |
| IR3 | 11,121,434 | 9,363,107 | 1,826,242 |
| IR4 | 10,265,940 | 8,643,715 | 1,686,327 |

**Supplemental** **Table II.** Quantitative polymerase chain reaction primers

| **Genes** | **Forward primers (5'-3')** | **Reverse primers (5'-3')** |
| --- | --- | --- |
| NOS2 | CACCAAGCTGAACTTGAGCGA | CCATAGGAAAAGACTGCACCGA |
| IL1β | GCAACTGTTCCTGAACTCAACT | ATCTTTTGGGGTCCGTCAACT |
| IL6 | TAGTCCTTCCTACCCCAATTTCC | TTGGTCCTTAGCCACTCCTTC |
| TNFα | AAGCCTGTAGCCCACGTCGTA | GGCACCACTAGTTGGTTGTCTTTG |
| Arg1 | CTGAGAAACGGAACCGCGA | TGCTCTTTGATCTGGCGGA |
| MRC1 | TTCAGCTATTGGACGCGAGG | GAATCTGACACCCAGCGGAA |
| Fizz1 | CCAATCCAGCTAACTATCCCTCC | CCAGTCAACGAGTAAGCACAG |
| IL10 | GCTCTTACTGACTGGCATGAG | CGCAGCTCTAGGAGCATGTG |
| STAT1 | GCCGAGAACATACCAGAGAATC | GATGTATCCAGTTCGCTTAGGG |
| CCL2 | TTAAAAACCTGGATCGGAACCAA | GCATTAGCTTCAGATTTACGGGT |
| CCL4 | TTCCTGCTGTTTCTCTTACACCT | CTGTCTGCCTCTTTTGGTCAG |
| CXCL1 | CTGGGATTCACCTCAAGAACATC | CAGGGTCAAGGCAAGCCTC |
| CXCL2 | CCAACCACCAGGCTACAGG | GCGTCACACTCAAGCTCTG |
| CD36 | TCTCCTAGTAGGCGTGGGTC | CACGGGGTCTCAACCATTCA |
| CD64 | GCAAGTTAGAAGCGATGGCG | ATGCCATGGTCCCACAGTTT |
| Dynlt1a | CCAGCACAGCAAAGTCAACC | GCAAGGTGGTCAGATGGACA |
| Prdx2 | GCAAATCGGAAAGTCGGCTC | CTCCGTGGGGCAAACAAAAG |
| Tubb5 | TGCCCTTTGTCCTCCAGTTT | TAGGAAGCACAGGGTAACAACC |
| Pri-mir155 | GGCTTGCTGAAGGCTGTATG | TGGACTTGTCATCCTCCCAC |
| β-actin | ACCTTCTACAATGAGCTGCG | CTGGATGGCTACGTACATGG |
| miR-155-5p | ACGCGCGTTAATGCTAATTGTGATAGGGGT | Universal reverse primer |
| miR-484 | TCAGGCTCAGTCCCCTCCC | Universal reverse primer |
| miR-novel | CGGCGAGGGGAATCTGACTGTCT | Universal reverse primer |
| miR-9-5p | CCGGGCGTCTTTGGTTATCTAGCTGTATGA | Universal reverse primer |
| miR-151-3p | ACGCTAGACTGAGGCTCCTTGAGG | Universal reverse primer |
| Mouse U6 | TGGCCCCTGCGCAAGGATG | Universal reverse primer |

**Supplemental** **Table III.** Differential expressed miRNAs

| **miRNAs** | **Fold change** | **P value** | **Up/Down** |
| --- | --- | --- | --- |
| mmu-miR-novel-chr7_40384 | 35 | 0.000009 | Up |
| mmu-miR-novel-chr7_40362 | 23.25 | 0.000284 | Up |
| mmu-miR-155-5p | 20.75 | 0.036441 | Up |
| mmu-miR-484 | 20.5 | 0.000000 | Up |
| mmu-miR-132-3p | 14.75 | 0.005908 | Up |
| mmu-miR-7a-5p | 7.96226 | 0.000934 | Up |
| mmu-miR-652-3p | 4.61261 | 0.028089 | Up |
| mmu-miR-15b-5p | 4.26087 | 0.036146 | Up |
| mmu-miR-181d-5p | 3.98947 | 0.035234 | Up |
| mmu-miR-423-5p | 3.29851 | 0.038472 | Up |
| mmu-miR-9-5p | -39.75 | 0.000194 | Down |
| mmu-miR-novel-chr7_40665 | -30.75 | 0.049948 | Down |
| mmu-miR-novel-chr11_6439 | -7.772201 | 0.009685 | Down |
| mmu-miR-122-5p | -6.764706 | 0.04945 | Down |
| mmu-miR-novel-chr5_35529 | -3.401961 | 0.049435 | Down |
| mmu-miR-151-3p | -2.64407 | 0.028899 | Down |
| mmu-miR-328-3p | -2.042035 | 0.024533 | Down |

**Supplemental** **Figures**

Supplemental Figure 1. IR-EVs didn’t enhance the expression of proinflammatory factors including (A) IL1β and (B) IL6 in circulating leukocytes, but decreased the expression of (C) IL10 in circulating leukocytes of IR-injured mice. ^*^, P <0.05; ^**^, P <0.01.


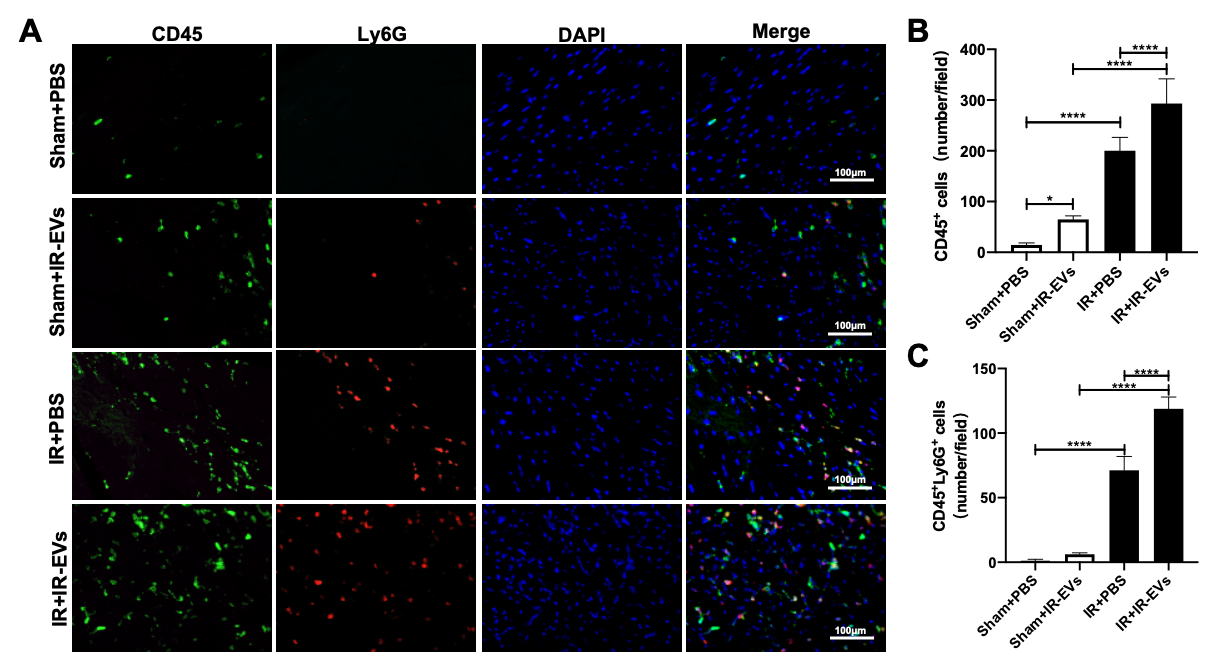


Supplemental Figure 2. Immunofluorescence staining of CD45 and Ly6G in the heart tissues. IR-EV transfusion promoted the infiltration of CD45^+^ inflammatory cells as well as CD45^+^Ly6G^+^ neutrophils in the IR-injured heart.

Supplemental Figure 3. GW4869 downregulated the Incremental EVs induced by IR injury in the heart. Intraperitoneal injection with GW4869 1h before IR surgery reduced the EV production in the heart.


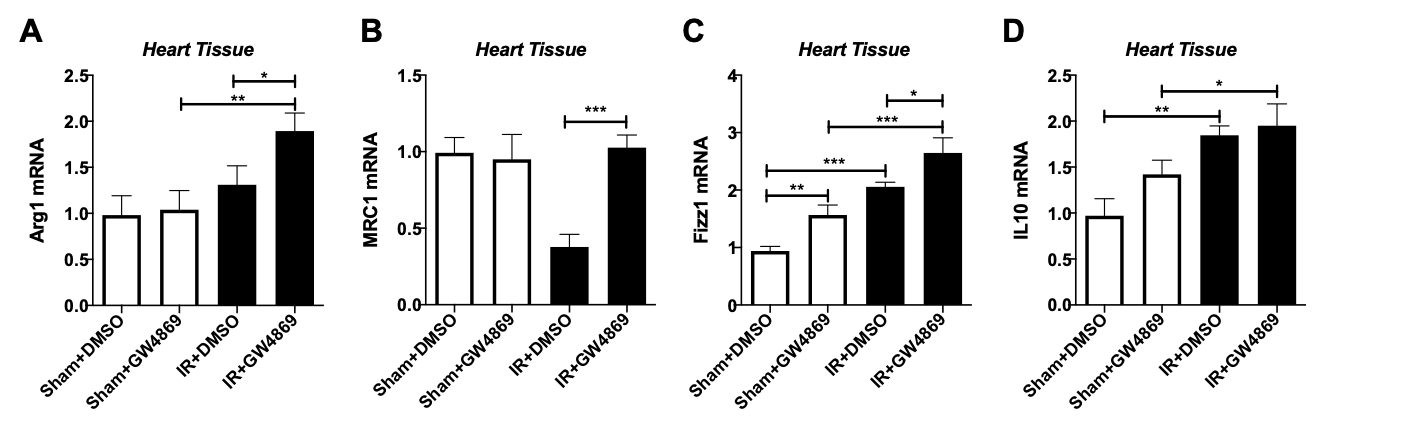


Supplemental Figure 4. GW4869 modulated the expression of macrophage polarization related genes in IR-injured heart. GW4869 treatment significantly increased the expression of M2-polarization related genes (A) Arg1, (B) MRC1, and (C) Fizz1. However, (D) IL10 were not significantly up-regulated in IR mice treated with GW4869 compared with IR mice. ^*^, P <0.05; ^**^, P <0.01; ^***^, P <0.001; ^****^, P <0.0001.

Supplemental Figure 5. IR-EVs regulated the expression of macrophage polarization related genes in macrophages. With the increase of IR-EVs stimulation time, (A) the NOS2 expression of macrophages was enhaced, while (B) the IL10 expression was decreased. (C) The expression of proinflammatory factors peaked when macrophages were stimulated with IR-EVs for 3h. (D) Significantly increased release of IL6 in cell culture media was found after IR-EV stimulation for 6h .^*^, P <0.05; ^**^, P <0.01; ^***^, P <0.001; ^****^, P <0.0001.


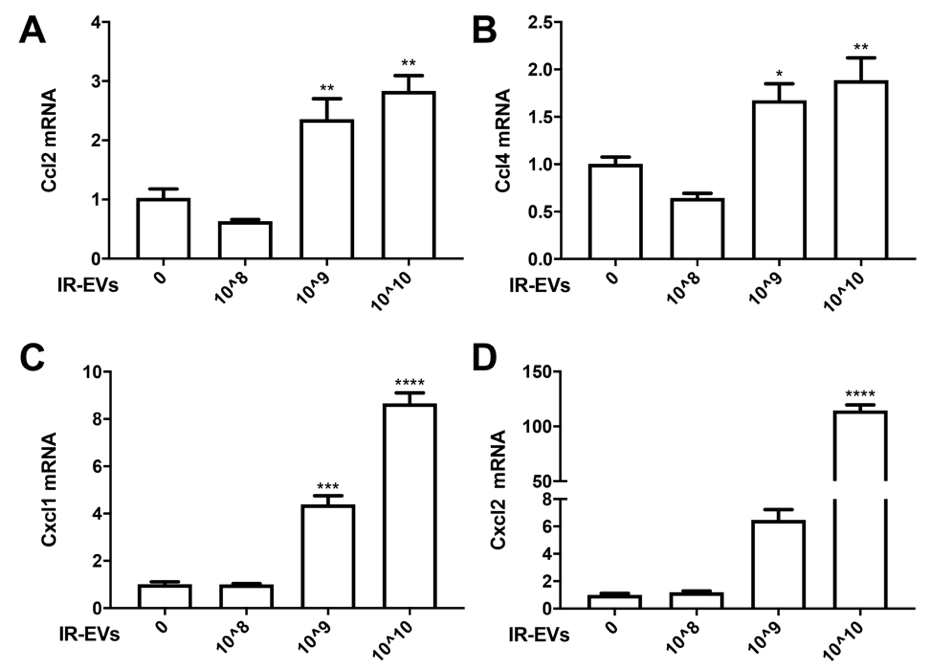


Supplemental Figure 6. Effects of different IR-EVs concentrations on chemokine expression of macrophages. The expression of (A) Ccl2, (B) Ccl4, (C) Cxcl1 and (D) Cxcl2 in macrophages treated with different concentrations of IR-EVs (0, 10^8^, 10^9^, and 10^10^ /mL) for 12 h. ^*^, P <0.05; ^**^, P <0.01; ^***^, P <0.001; ^****^, P <0.0001.

Supplemental Figure 7. The expression of selected miRNAs in the circulating EVs. Compared with circulating S-EVs, circulating IR-EVs enriched higher levels of (A) miR-155-5p, but lower levels of (B) miR-9-5p and (C) miR-151-3p. ^*^, P <0.05; ^**^, ^***^, P <0.001; ^****^, P <0.0001.

Supplemental Figure 8. The proinflammatory effect of IR-EVs in the brain tissues. (A) Myocardial IR led to increased release of EVs in the circulation. (B) Myocardial IR injury induced increased expressions of IL1β and Cxcl1 in the brain tissues. (C) GW4869 treatment didn’t significantly influence the expressions of proinflammatory genes induced by myocardial IR in the brain tissues. (D) Bioluminescence imaging showed the lingering of IR-EVs in the brain tissues 24h after intravenous injection of DiR-labelled EVs. (E) The expression of proinflammatory genes in the brain tissues 24h after intravenous injection of IR-EVs or PBS. *, P <0.05; ***, P <0.001;

Supplemental Figure 9. The recruitment of inflammatory cells in various organs by IR-EV injection. Intravenous administration of IR-EVs promoted the recruitment of inflammatory cells in various organs especially lung, liver and kidney.


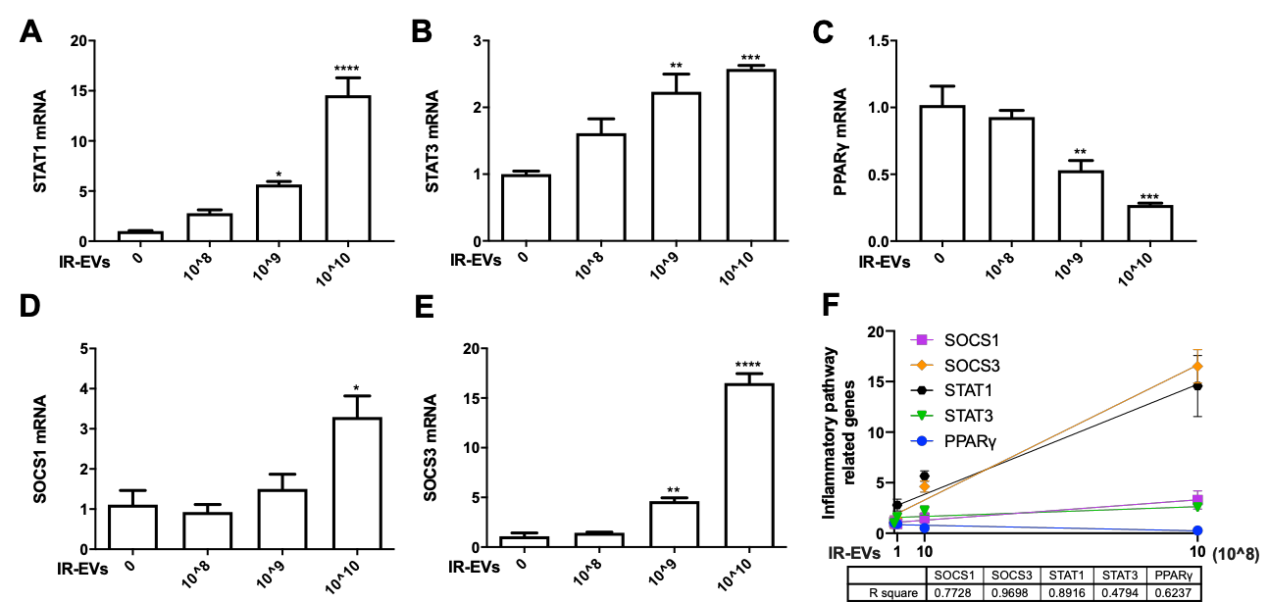


Supplemental Figure 10. The expression of inflammatory pathway genes in IR-EV treated macrophages. The expression of (A) STAT1, (B) STAT3, (C) PPARγ, (D) SOCS1, and (E) SOCS3 in macrophages treated with different concentrations of IR-EVs (0, 10^8^, 10^9^, and 10^10^ /mL) for 24 h. (F) Positive correlations between IR-EV concentration and the expression of inflammatory pathway related genes in macrophages treated with IR-EVs for 24 h. ^*^, P <0.05; ^**^, P <0.01; ^***^, P <0.001; ^****^, P <0.0001.

**
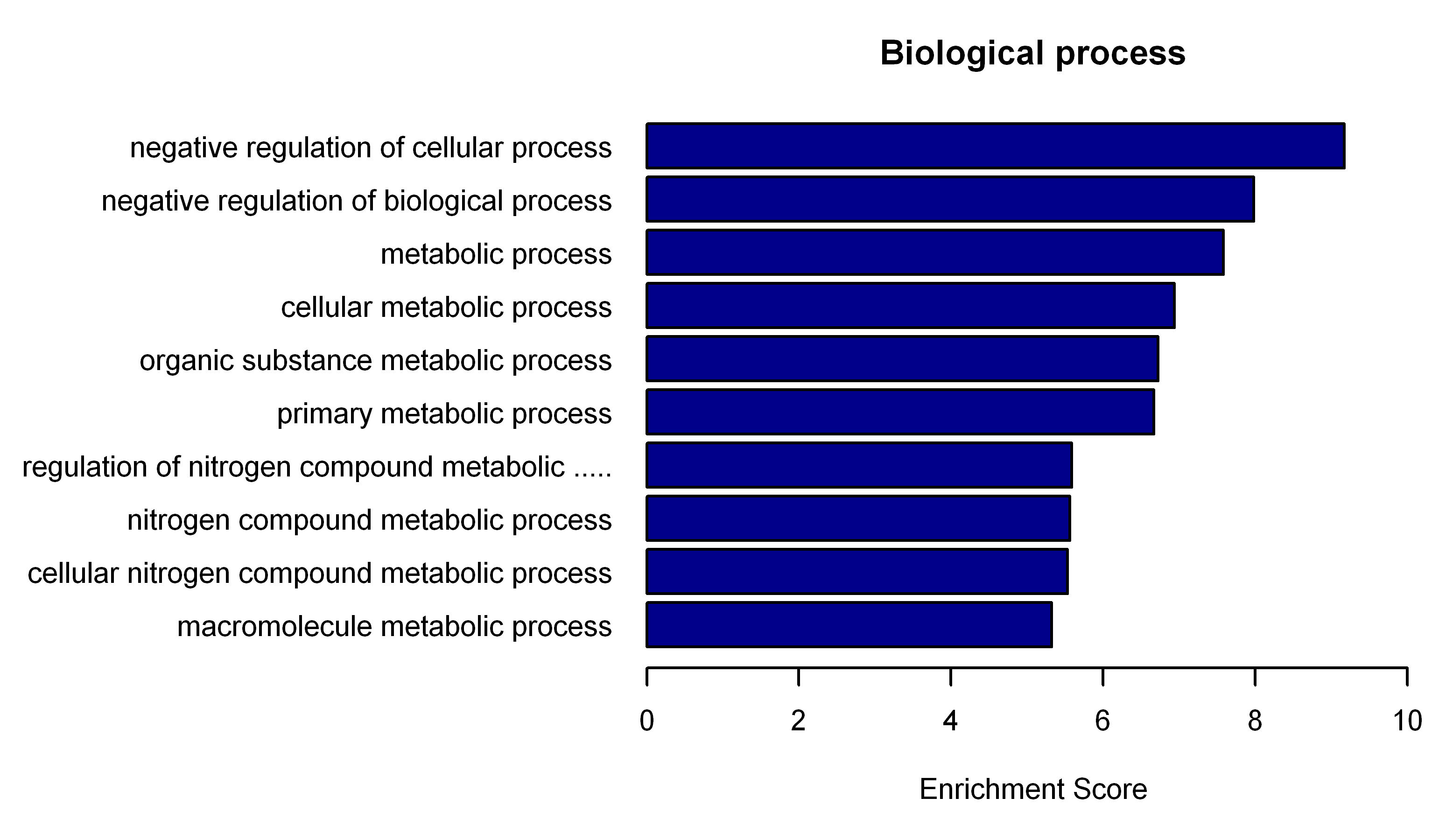
**

Supplemental Figure 11. Biological process prediction of up-regulated miRNAs

**
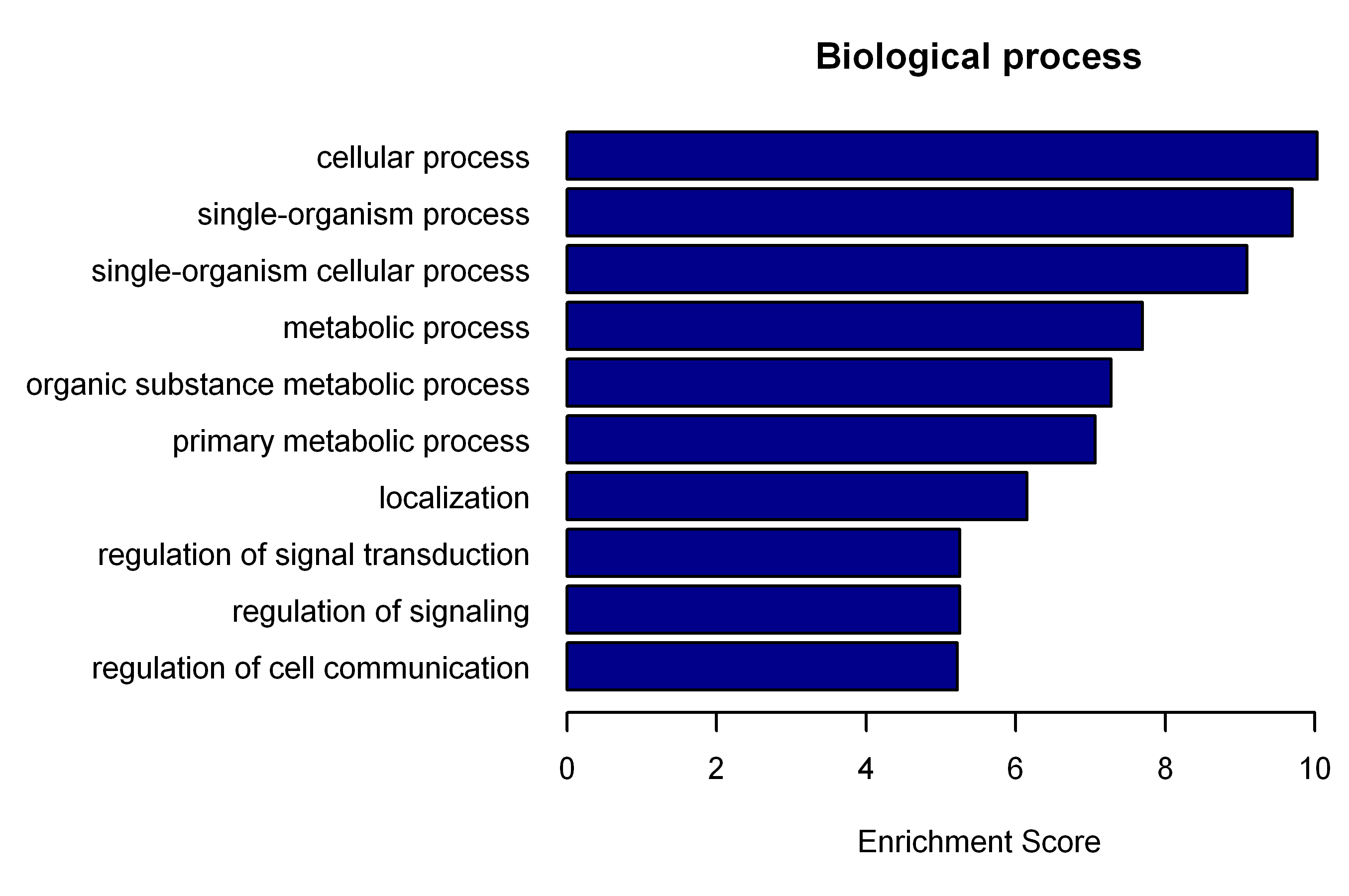
**

Supplemental Figure 12. Biological process prediction of down-regulated miRNAs.
